# Targeting Energy Metabolism in Cancer Treatment

**DOI:** 10.3390/ijms23105572

**Published:** 2022-05-16

**Authors:** Joanna Kubik, Ewelina Humeniuk, Grzegorz Adamczuk, Barbara Madej-Czerwonka, Agnieszka Korga-Plewko

**Affiliations:** 1 Independent Medical Biology Unit, Faculty of Pharmacy, Medical University of Lublin, 20-093 Lublin, Poland; joannakubik@umlub.pl (J.K.); grzegorzadamczuk@umlub.pl (G.A.); agnieszkakorga@umlub.pl (A.K.-P.); 2 Human Anatomy Department, Faculty of Medicine, Medical University of Lublin, 20-090 Lublin, Poland; barbara.madej-czerwonka@umlub.pl

**Keywords:** cancer metabolism, cancer treatment, glycolysis, oxidative phosphorylation

## Abstract

Cancer is the second most common cause of death worldwide after cardiovascular diseases. The development of molecular and biochemical techniques has expanded the knowledge of changes occurring in specific metabolic pathways of cancer cells. Increased aerobic glycolysis, the promotion of anaplerotic responses, and especially the dependence of cells on glutamine and fatty acid metabolism have become subjects of study. Despite many cancer treatment strategies, many patients with neoplastic diseases cannot be completely cured due to the development of resistance in cancer cells to currently used therapeutic approaches. It is now becoming a priority to develop new treatment strategies that are highly effective and have few side effects. In this review, we present the current knowledge of the enzymes involved in the different steps of glycolysis, the Krebs cycle, and the pentose phosphate pathway, and possible targeted therapies. The review also focuses on presenting the differences between cancer cells and normal cells in terms of metabolic phenotype. Knowledge of cancer cell metabolism is constantly evolving, and further research is needed to develop new strategies for anti-cancer therapies.

## 1. Introduction

Cancer is the second leading cause of death worldwide after cardiovascular diseases with over 9.5 million cases accounting for 13% of all deaths [1]. Despite the multiple cancer treatment strategies, such as chemotherapy, radiotherapy, immunotherapy, and surgery, the patients who suffer from cancer cannot be completely treated. In addition, the resistance to prior therapy becomes the greatest challenge for successful cancer treatment. Currently, the development of anti-cancer strategies that are highly effective and have few side effects is a high priority [2]. Learning about the biology of cancer and disturbances in metabolic pathways, which are a hallmark of cancer cells, allows learning about new therapeutic targets. Over the last century, there has been increasing interest in cancer energetic metabolism [3]. The metabolism of cancer cells is highly different from normal cells. It depends on many factors, e.g., the microenvironment of the tumor cells as well as numerous mutations occurring during cancerogenesis and progression of the disease. Moreover, the cancer cells themselves modify their microenvironment to adapt to unfavorable development conditions [4,5]. Thus, targeting the metabolisms of cancer cells may have an enormous impact on the effectiveness of the therapy used. Otto Warburg was the first scientist who started research focused on the metabolic processes in cancer cells, mostly concentrating on cellular respiration. He has noted that tumor cells as compared to normal cells promote glycolysis instead of mitochondrial respiration, even in the presence of oxygen. This phenomenon is today known as the Warburg effect or oxygen glycolysis. Initially, Warburg claimed that oxygen glycolysis in cancer cells is a result of irreversibly dysfunctional mitochondria and is necessary to initiate cancer transformation [6,7]. The role of oxygen glycolysis is to support the proliferation of cancer cells, the induction of mesenchymal transition, promote metastasis, modulate the pH and tumor microenvironment, and affect other metabolic pathways [8,9,10]. For example, as a result of the local acidification of the microenvironment, the expression of vascular endothelial growth factor A (VEGF-A) increases, which increases the proliferation of cancer cells [11]. The glycolysis pathway in cancer cells can deliver ATP up to 100 times faster than the tricarboxylic acid cycle (TCA) and oxidative phosphorylation (OXPHOS) in mitochondria because the production of lactate from glucose is faster than the total oxidation of glucose in the mitochondria [12,13]. During the changes in the tumor microenvironment to increase the demand for ATP, there was an increase in the number of ATP-dependent membrane pumps. As a consequence, oxygen glycolysis increased sharply, while the process of oxidative phosphorylation remained constant [14].

Contrary to what Warburg claimed, other scientists noted that the decrease in mitochondrial activity might be a result of increased glycolysis. Sidney Weinhouse undermined the Warburg hypothesis by performing isotope labeling. He stated that the speed of oxidative phosphorylation both in normal cells and cancer cells is similar, which indicates that the mitochondria of both cells work properly [15]. Unfortunately, today there is no clear position on this matter, but it is known that Warburg’s initial hypothesis that cancer results from impaired mitochondrial metabolism was proved to be incorrect. However, we still observe increased glycolysis in tumors, even in the presence of oxygen, which has become the subject of further research into the metabolism of cancer cells [16].

Recent studies indicate that cancer is characterized by metabolic heterogeneity due to the existence of metabolic cooperation. Therefore, a new hypothesis on cancer metabolism has been proposed as the reverse Warburg effect. It consists of the cooperation between stromal fibroblasts and cancer cells to meet their metabolic needs. It assumes that the Warburg effect occurs in normal cells mainly in the stromal fibroblasts, not in cancer cells [17,18]. Reactive oxygen species generated in the cancer cells move towards the fibroblast stromal cells associated with cancer (CAF) and induce oxidative stress [19]. This leads to the activation of hypoxia-inducible factor 1 (HIF-1α) which is the main regulatory subunit of HIF and kappa light chain enhancer of activated B cells (NFκB) [20]. Catabolic processes also take place in the stromal cells, which provide highly energetic compounds, especially lactate, pyruvate, ketone bodies, and fatty acids, which are used for anabolic reactions performed by cancer cells [21,22]. As a result, cancer cells can carry out the process of aerobic respiration in the mitochondria, which consequently leads to the production of an increased amount of ATP, which is used for the proliferation of cancer cells and promotes tumor growth. Metabolic cooperation between these cells helps to react quickly to changes in nutrient availability, so that maximum cell proliferation and growth are maintained [23].

For this reason, targeting the metabolism of tumor cells, different from normal cells, seems to be a safe, highly effective option for cancer treatment [24,25]. In addition, despite the possibility of cancer cells changing their metabolism during treatment, targeting it may re-sensitize tumors for prior ineffective therapies [26]. This review summarizes the current knowledge on induction of metabolism disturbance of tumor cells as a cancer treatment and focuses on presenting the differences affecting the metabolic phenotype between cancer and normal cells.

## 2. Glycolysis Pathway and Therapeutic Targets

### 2.1. Glucose Transporters

The glycolytic phenotype of cancer cells is associated with overexpression of glucose transporters (GLUTs) which leads to increased glucose utilization as compared to normal cells [27]. Therefore, an effective anti-cancer approach may be reducing the availability of glucose to cancer cells. It was noted that patients on a restricted carbohydrate diet showed therapeutic benefits. To obtain the desired therapeutic effect, attempts have been made to block glucose transporters [28,29].

Several compounds show positive activity in preclinical/clinical studies, however, selective blocking of GLUT is difficult due to the fact that GLUT is present in all cells. GLUT inhibitors have been isolated from natural products as well as synthetically.

**Figure 1:**
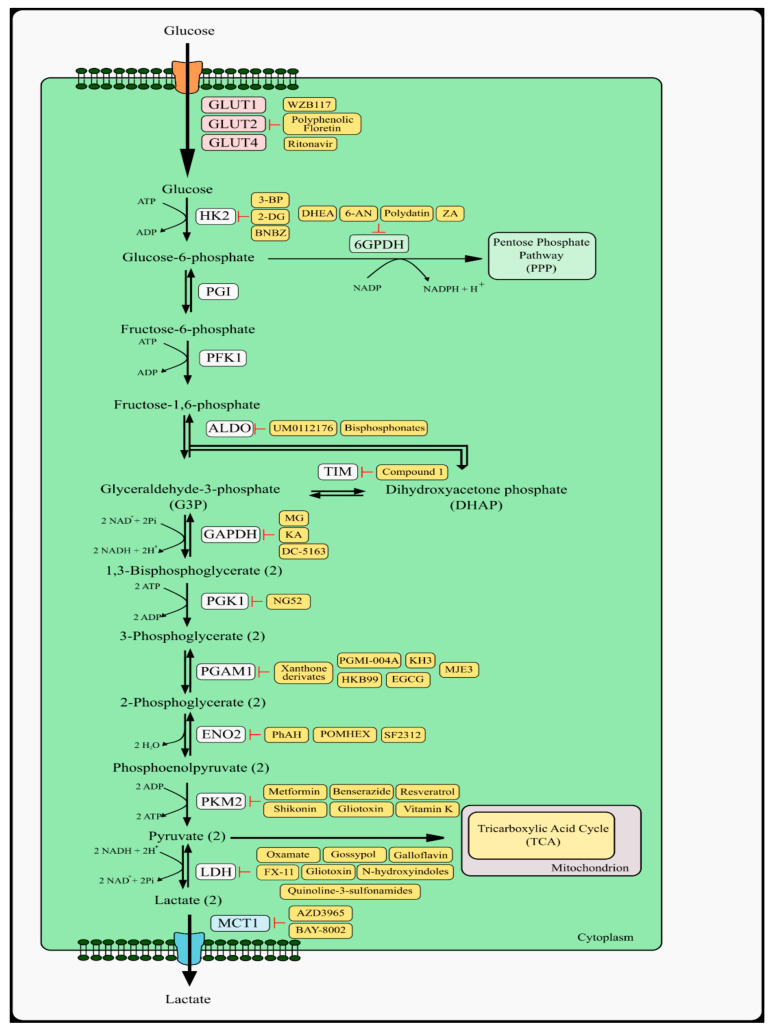
Graphical representation of inhibitors of glucose transport, glycolysis pathway, 6GPDH, and its metabolic target. GLUT—glucose transporter; HK2—hexokinase type 2; 2-DG—2-deoxyglucose; 3-BP—3-bromopyruvate; BNBZ—benitrobenrazide; DHEA—dehydroepiandosterone; 6-AN—6-aminonicotinamide; ZA—zoledronic acid; 6GPDH—glucose-6-phosphate dehydrogenase; PGI—phosphoglucose isomerase; PFK1—phosphofructokinase 1; ALDO—aldolase; TIM—triosephosphate isomerase; GAPDH—glyceraldehyde 3-phosphate dehydrogenase; KA—koningic acid; MG—methylglyoxal; PGK1—phosphoglycerate kinase 1; PGAM1—phosphoglycerate mutase 1; EGCG—epigallocatechin-3-gallate; ENO2—γ-enolase; PK—pyruvate kinase; LDH—lactate dehydrogenase; MCT1—monocarboxylate transporter 1.

An example is the GLUT1 inhibitor 2-Fluoro-6-(m-hydroxybenzoyloxy) phenyl m-hydroxybenzoate (WZB117) which inhibits the growth of lung cancer cells (A549) and breast cancer cells (MCF7) and has synergistic effects with cisplatin and paclitaxel [30]. After administration of WZB117, there was an immediate reduction in glucose transport in tumor cells, which led to a decrease in the activity of glycolytic enzymes, the stress in the endoplasmic reticulum (ER), and a decrease in ATP and lactate levels. As a consequence of changes in the energy homeostasis of the neoplastic cell, the cell cycle was inhibited, as was the growth of cancer cells (Figure 1) [31,32].

Polyphenolic phloretin (Ph) is a compound isolated from apple and acts as a GLUT2 antagonist in triple-negative breast cancer (TNBC). Interestingly, it was found that Ph inhibited cell growth and arrested the cell cycle in MDA-MB-231 cells in a p53 mutant-dependent manner [33,34,35]. Other studies have shown that Ph inhibits the growth of colon cancer cells (COLO205) [36]. GLUT2 activates p53 signaling as a protein involved in cell cycle control and apoptosis [37]. Ritonavir, a protease inhibitor that is an FDA-approved anti-viral drug, inhibits GLUT4 in multiple myeloma (MM) [38,39].

### 2.2. Hexokinase Type 2 and Phosphoglucose Isomerase

In cancer cells, the expression of glycolytic enzymes is often changed [15]. It is suggested that the resulting intermediate metabolites glucose-6-phosphate (G6P), phosphoenolpyruvate (PEP), 3-phosphoglycerate (3PG), 2-phosphoglycerate (2PG), and lactate during oxygen glycolysis may play a similar role to oncogenes, resulting in a change in signaling pathways and blocking cell differentiation [37]. Additionally, increased expression of the enzyme leads to increased proliferation of neoplastic cells [40]. In the first stage of glycolysis, hexokinase (HK), especially the hexokinase type 2 (HK2) isoform converting glucose into G6P, shows an increased level of expression, which leads to an increased level of glycolysis [41]. 

One of the anti-cancer treatment strategies connected with the glycolysis pathway is targeting the enzyme HK2, which converts glucose into G6P and is responsible for the first step of glycolysis. HK2 is upregulated in many cancer cells [42].

Two HK2 inhibitors have been identified, 2-deoxyglucose (2-DG) and 3-bromopyruvate (3-BP). 2-DG is a glucose molecule in which the 2-hydroxyl group is replaced with hydrogen and therefore cannot enter the glycolysis pathway [43,44]. 2-DG acts as a glucose mimetic by inhibiting the formation of G6P from glucose, leading to a reduction in ATP production and subsequent cell death. There is phosphorylation of 2-deoxyglucose to 2-deoxyglucose-6-phosphate, which cannot be further metabolized [45,46]. In vitro studies showed that 2-DG inhibits the growth of many cancer cell lines [47]. 2-DG has undergone a phase I/II clinical trial in the treatment of solid tumors and the treatment of hormone-refractory prostate cancer, but tumor growth and toxicity eliminated it from further studies (NCT00633087).

Preclinical studies showed that 3-BP inhibits HK2 and is a promising anti-cancer drug targeting glycolysis. Over the past 20 years, 3-BP has been studied in many in vitro and in vivo studies on several types of cancer. The promising anti-cancer results gave hope for the continuation of research on 3-BP in clinical trials. Unfortunately, no clinical trials using 3-BP in the treatment of neoplastic diseases have been approved to date [48,49,50,51]. Based on individual literature reports on the use of 3-BP in volunteer patients and previous preclinical research, several problems are mentioned related to the use of 3-BP in clinical practice. These include, for example, accelerated metabolism of 3-BP induced by glutathione, rapid attachment of 3-BP to serum proteins, burning sensation in the veins during intravenous infusion of 3-BP, and resistance of glutathione (GSH)-rich tumor cells. One of the proposed solutions to many problems related to the use of 3-BP in clinical practice proposed by El Sayed et al. could be synthesizing PEGylated 3-BP. Among other things, this could prevent 3-BP from binding to serum proteins [52,53,54]. New small-molecule compounds with the potential to inhibit HK2 activity in cancer cells are constantly being sought. One of the recently investigated compounds with the ability to inhibit HK2 is benitrobenrazide (BNBZ). The conducted in vitro research revealed that BNBZ causes suppression of proliferation and induction of apoptosis in pancreatic cancer cells with overexpression of HK2 (SW1990) through inhibition of this enzyme. These results were confirmed in in vivo studies using the SW1990 and SW480 xenograft model, where a significant reduction in tumor mass was noted after oral administration of BNBZ at a dose of 75 and 150 mg/kg. It is promising that preliminary in vivo studies have shown low toxicity of BNBZ, making it a good candidate for further extended research [55,56].

Considering the next two enzymes involved in the glycolysis pathway, i.e., glucose-6-phosphate isomerase and phosphofructokinase 1 (PFK1), no reports of potential inhibitors of these enzymes in the field of cancer cell metabolism have been found to date. The lack of specific inhibitors of these enzymes is an open path to the search for new molecules with potential anti-tumor activity. Glucose-6-phosphate, also known as phosphoglucose isomerase (PGI), is an extremely interesting enzyme that, apart from glycolysis, is involved in the process of glucose synthesis and the pentose phosphate pathway [57]. Research has shown that PGI has strong cytokine-like effects and also functions as such molecules as autocrine motility factor (AMF), neuroleukin (NLK), and maturation factor (MF). Despite the fact that PGI, AMF, NLK, and MF are encoded by one gene and have the same structure, each of them has different assigned functions [58,59,60].

### 2.3. Aldolase

The fourth enzyme involved in the glycolysis process is aldolase, which has three isoenzymes: aldolase A (ALDOA), aldolase B (ALDOB), and aldolase C (ALDOC). Isoenzymes differ mainly in the level of expression in various tissues. Many studies have shown high expression of ALDOA in cell lines and patient samples, including lung cancer, liver cancer, colon cancer, and osteosarcoma [61,62,63,64,65]. The importance of different levels of ALDOB expression in tumors is controversial due to the discrepancies between the different types of cancer. In colorectal adenocarcinoma, ALDOB overexpression was observed during the epithelial–mesenchymal transition and was associated with poorer survival [65]. However, there are also reports of a low ALDOB level in gastric cancer, which is associated with a poor prognosis for patients [66]. UM0112176 is an inhibitor of ALDOA which was verified in in vitro studies by Gizak et al. [67]. The use of the compound in both tumor and normal cells resulted in the accumulation of substrate and a decrease in the amount of products of ALDOA reaction. The studies also showed that 10 µM of UM0112176 significantly inhibited the proliferation of cancer cell lines such as non-small cell lung cancer and pancreatic adenocarcinoma, without cytotoxic effect on normal human cell lines. Even though UM0112176 inhibits the metabolic function of ALDOA, its cytotoxic effect on cancer cells is related to the inhibition of the non-metabolic functions of this enzyme mainly by disturbing the interaction of ALDOA with F-actin. It has been proven that overexpression of ALDOA has an influence on the preservation of the integrity of the cytoskeleton of tumor cells during the epithelial–mesenchymal transition [67].

Another group of ALDOA inhibitors is bisphosphonate-based inhibitors. Among the compounds tested by Heron et al., 2-phosphate-naphthalene-6-bisphosphonate (compound 2) showed cytotoxic activity towards HeLa cells [68].

### 2.4. Glyceraldehyde 3-Phosphate Dehydrogenase and Phosphoglycerate Kinase 1

The next enzyme in the glycolysis pathway is glyceraldehyde 3-phosphate dehydrogenase (GAPDH). Several compounds have been identified, including koningic acid (KA), methylglyoxal (MG), and DC-5163. Koningic acid (KA, heptelidic acid) is a sesquiterpene metabolite which is an irreversible GAPDH inhibitor first isolated in 1980 from strains of several species of fungi: * Anthostoina avocetta*, *Chaetomium globosum*, *Trichoderma viride*, and *Gliocladicon virens* [69,70]. Liberti et al. [71] conducted in vitro experiments on a set of sixty different cancer cell lines and different sensitivity to KA was found at the concentration of 10 µM. The study showed a differential response to KA among cell lines representing the same type of cancer, for example, the most resistant cell lines were MCF-7 (breast cancer), NCI-H226 (lung cancer), and UACC-257 (melanoma), while the most sensitive were SK-MEL28, UACC-62, SK-MEL2, M14, SK-MEL5 (melanoma), NCI-H522 (lung cancer), and BT-549 (breast cancer). In the case of breast cancer, the studies were confirmed in in vivo studies using both KA-resistant and -sensitive cell lines in orthotopic breast cancer models. It was found that KA is well tolerated by laboratory animals at a dose of 1 mg/kg body weight, which resulted in a circulating plasma concentration of 0.7 µM [71]. Other conclusions can be drawn from the studies conducted by Rahier et al. [72], which show that KA is a non-selective anti-cancer compound and shows poor activity in vivo in the A549 lung carcinoma xenograft model [72]. KA as a compound with anti-cancer potential requires further, in-depth research due to various scientific reports.

Another GAPDH inhibitor is methylglyoxal, which is a physiologically occurring metabolite of glucose metabolism. The inhibition of GAPDH by methylglyoxal through the glycation mechanism has been proven in experimental studies using rabbit GAPDH by Lee et al. [73]. One of the recently synthesized small-molecule GAPDH inhibitors is (3-benzyl-N-[3-chloro-4-methoxyphenyl] imidazolidine-1-carbothioamide (DC-5163). DC-5163 inhibits GAPDH activity in five tumor cell lines including breast cancer, colorectal cancer, and lung cancer. Further studies using the MDA-MB-231 breast cancer line showed that DC-6163 inhibits proliferation, decreases glucose consumption and lactate production, as well as induces apoptosis. At the same time, DC-5163 did not affect the proliferation of normal human breast epithelial cells [74]. This molecule may be an interesting alternative in anti-cancer therapy, but further research is needed using other types of cancer and an in vivo model. 

The next enzyme in the glycolysis pathway is phosphoglycerate kinase 1 (PGK1). NG52 is a small molecule compound that possesses the ability to inhibit PGK1. The anti-cancer activity of this compound has been proven in the studies carried out by Wang et al. [75] on glioma cells. NG52 inhibits the proliferation of U87 and U251 glioma cell lines as well as primary glioma patients’ samples. Results obtained in in vitro studies were confirmed in an in vivo glioma patient-derived xenograft model [75]. 

### 2.5. Phosphoglycerate Mutase 1

A glycolysis enzyme with an extensively studied group of inhibitors with potential anti-cancer activity is phosphoglycerate mutase 1 (PGAM1) [76]. These inhibitors include PGMI-004A, HKB99, MJE3, epigallocatechin-3-gallate (EGCG), xanthone derivatives, and KH3. Hitosungi et al. [77] designed and tested PGMI-004A, the alizarin Red S derivative, which directly inhibits the enzymatic activity of PGM1. In performed in vitro tests, PGMI-004A showed cytotoxic activity towards many cancer cell lines including lung cancer, head and neck cancer, and leukemias. Simultaneously, it did not affect the proliferation of human skin and foreskin fibroblasts, as well as melanocytes and keratinocytes. In in vivo studies, a well-tolerated dose of PGMI-004A was established at the level of 100 mg/kg/day. In an experiment using mouse lung cancer xenografts, the anti-tumor activity of PGMI-004A was confirmed by the inhibition of tumor growth and the inhibition of PGAM1 activity in resected tumors [77].

Another alizarin Red S derivative which is an allosteric inhibitor of PGAM1 is HKB99. An in vitro study of the effect of HKB99 on non-small cell lung cancer (NSCLC), cells showed inhibition of proliferation and migration of cells and induction of apoptosis. Moreover, in vitro and in vivo studies with the use of xenografts have shown that HKB99 enhances the cytotoxic effect of erlotinib on NSCLC cells and overcomes the resistance to erlotinib [78,79]. One of the listed PGAM1 inhibitors is MJE3, but there are very limited scientific reports about its anti-cancer activity. Evans et al. [80] showed that MJE3 inhibits the proliferation of MDA-MB-231 breast cancer cells by inhibiting the activity of PGAM1 [80].

The group of compounds constituting potent PGAM1 inhibitors includes xanthone derivatives which were synthesized and tested on representative lung cancer, breast cancer, and pancreatic cancer cell lines by Wang et al. [81]. Xanthone derivates showed a stronger inhibitory effect on PGAM1 activity compared to PGMI-004A, however, most of the derivatives demonstrated cytotoxic activity towards tested cell lines comparable to PGMI-004A. The suspected reason is the poor permeability of xanthone derivatives through biological membranes [81].

The next PGAM1 inhibitor is KH3, which has shown anti-tumor activity in vitro and in vivo in pancreatic ductal adenocarcinoma [82]. The last PGAM1 inhibitor is epigallocatechin-3-gallate (EGCG), which is an organic chemical compound from the group of flavonoids derived from catechin. It is found in significant amounts in green tea extracts. EGCG effectively inhibited PGAM1 in the form of a liposome that had a greater ability to penetrate through the cell membrane in H1299 lung cancer cells [83]. None of the PGAM1 inhibitors has entered the phase of clinical trials. Currently, work is underway to find other inhibitors of PGAM1, especially natural, inter alia, using in silico methods [84]. Another enzyme involved in the glycolytic pathway catalyzing the reversible conversion of 2-phosphoglycerate to phosphoenolpyruvate is enolase. There are three isotypes of this enzyme: α-enolase (ENO1), β-enolase (ENO3), and γ-enolase (ENO2) [85]. ENO1 is overexpressed in many types of cancer [86]. However, a very interesting direction of research in the area of anti-cancer treatment in the context of enolase is the use of ENO2 inhibitors in tumors that have an ENO1 deletion. Muller et al. [87] identified the presence of homozygous deletions at the 1p36 locus corresponding to ENO1 in two glioblastoma cell lines, D423-MG22 and Gli56. In glioblastoma cell lines where an ENO1 deletion has occurred, the reaction in the glycolysis pathway is catalyzed by ENO2. In performed research, they showed that ENO2 inhibitor phosphonoacetohydroxamate (PhAH) is selectively toxic to cells with the ENO1 deletion, while it has no effect on glioblastoma cells without the deletion [87]. Two other ENO2 inhibitors, POMHEX and SF2312, are also active on cell lines with ENO1 deletion [88,89].

### 2.6. Pyruvate Kinase

The final step in glycolysis is the irreversible conversion reaction of phosphoenolpyruvate (PEP) to pyruvate and ATP, which is catalyzed by pyruvate kinase (PK) that belongs to the transferase class of enzymes [90,91]. The PKM2 isoform activates the HIF-1 transcription factor, which promotes high glucose metabolism, by adjusting the range of ATP needed for tumor cell proliferation [92]. Moreover, both HIF-1 and PKM2 lead to the activation of gene expression, which is responsible for the adaptation of the cell to hypoxic conditions. Another role of PKM2 is to redirect glucose metabolism towards the pentose pathway and to protect cancer cells against oxidative stress by generating a reducing force in the form of nicotinamide–adenine dinucleotide phosphate (NADPH) necessary for the regeneration of glutathione [93,94,95,96].

The role of PKM2 as a therapeutic target in the treatment of neoplastic diseases, despite numerous in vitro and in vivo studies, remains controversial. Studies performed by Israelsen et al. [97] showed that PKM2 deletion accelerated the formation of breast tumors in mice with a BRCA1 deletion [97]. Furthermore, elderly PKM2-knockout mice often developed spontaneous multi-focal hepatocellular carcinoma (HCC) [98]. In vivo studies on a mouse model of pancreatic ductal adenocarcinoma showed that the deletion of PKM2 does not affect the formation or progression of this tumor [99]. An increase in PKM2 occurs in patients with metastatic cancer and also correlates with late-stage cancer [95,100]. Contrary to the previously cited reports, many studies show that inhibition of PKM2 with various substances can inhibit the growth of neoplastic cells and induce apoptosis by influencing the energy metabolism of the cell [101]. In addition, research shows that changes in PKM2 expression are associated with drug resistance in various cancers, therefore PKM2 is a potential target of anti-cancer therapy. Thus, targeting PKM2 may effectively improve the effectiveness of chemotherapeutic drugs [102]. Many researchers report that PKM2 is a promising target for cancer therapy but in-depth studies with precise determination of the expression level and functions of this enzyme in a particular type of cancer are required [103]. 

In recent years, many conducted types of research have brought knowledge about natural and synthetic PKM2 inhibitors that have potential in anti-cancer treatment. These include previously known drugs that were used for other diseases, such as metformin, natural substances, as well as newly synthesized molecules [102]. Metformin increases the sensitivity of osteosarcoma stem cells to cisplatin and bladder cancer cells to combination therapy (docetaxel, trastuzumab, pertuzumab) in preclinical research [104,105]. Studies have shown that metformin disrupts the HIF1α/PKM2 signaling pathway, initiating the process of apoptosis in gastric cancer cells and inhibiting the epithelial–mesenchymal transition in oral squamous cell carcinoma and cervical carcinoma cells [106,107,108]. Currently, over 100 different clinical trials are planned and ongoing using metformin alone and in combination with other chemotherapeutic agents in various types of cancer due to its high potential as an anti-cancer drug, mostly because of activation of AMPK. Another drug, routinely used in the treatment of Parkinson’s disease, that inhibits PKM2 activity is benserazide. Anti-tumor activity of benserazide related to PKM2 inhibition was observed in melanoma cells [109]. 

The group of PKM2 inhibitors also includes substances of natural origin, such as shikonin, resveratrol, and vitamin K, but also mycotoxin–gliotoxin. Shikonin is an active substance extracted from the *Lithospermum erythrorhizon*, which has, inter alia, anti-cancer properties. Shikonin’s potential as an anti-cancer drug is based on a multi-way mechanism of action that includes the inhibition of PKM2 [108,110]. Wang et al. [110] in in vitro and in vivo studies showed that the resistance of bladder cancer cells is associated with PKM2 overexpression. The combination of cisplatin and shikonin resulted in the inhibition of proliferation of bladder cancer cells and induction of apoptosis. Moreover, in vivo studies showed that the combination of these two compounds causes a reduction in tumor growth and inhibition of the formation of metastases in bladder cancer [110]. The anti-tumor activity of shikonin related to the inhibition of PKM2 has also been shown in studies involving such tumors as cholangiocarcinoma, Lewis lung carcinoma, melanoma, esophageal cancer, and hepatocellular carcinoma [111,112,113]. Another natural compound that reduces the expression of PKM2 in neoplastic cells is a natural phytoalexin, resveratrol. Decreased expression of PKM2 caused by resveratrol treatment results in increased ER stress and elevated levels of mitochondrial fission proteins which are involved in the induction of apoptosis [114]. Zhao et al. [115] showed that resveratrol induces apoptosis in melanoma cells through the downregulation of the Erk/PKM2/Bcl-2 axis [115].

The vitamin K family includes fat-soluble substances that play physiological roles in the human body. Two vitamin K analogs, vitamin K3 and vitamin K5, are suggested as potential anti-cancer agents. Studies by Chen et al. [116] have shown that vitamins K3 and K5 inhibit the expression of PKM2, which leads to the disruption of the glycolysis process and a reduction in the viability of HeLa cells [116]. Many in vitro and in vivo studies demonstrated the cytotoxic effect of vitamins K3 and K5 on neoplastic cells through different mechanisms of action. As a result, they represent a promising development direction in the field of therapeutic adjuvants in cancer therapy [117,118,119]. Another natural compound that inhibits the enzymatic and kinase activity of PKM2 by direct bonding to this enzyme is a mycotoxin formed as a metabolite of marine fungi, gliotoxin. Preliminary studies have shown that gliotoxin inhibits cell proliferation in cancer cell lines such as glioblastoma, acute promyelocytic leukemia, chronic myelogenous leukemia, non-small cell lung cancer, breast cancer, prostate cancer, and colon cancer [120]. Compound 3k (C3k) is a selective PKM2 inhibitor that shows anti-tumor activity in various types of cancer cells with high PKM2 expression such as colon cancer (HCT116) and cervical cancer (HeLa) [121].

## 3. Pyruvate and Its Further Fate

### 3.1. Lactate Dehydrogenase

Lactate dehydrogenase (LDH) is an enzyme belonging to the class of oxidoreductases. This enzyme is most frequently found in the form of LDHA and LDHB isoforms [122,123].

In preclinical studies, it has been shown that inhibition of LDHA may have potential application in cancer therapy. Inhibition of LDHA in cancer cells leads to a decrease in the potential of the mitochondrial membrane and leads to cell death [45,124]. Several LDHA inhibitors with different mechanisms of action have been identified. A compound that inhibits the LDHA and also LDHB action is oxamate. It is a pyruvate analog that competes for the enzyme binding site. Oxamate anti-tumor properties have been confirmed in an in vitro model of gastric cancer on SGC7901 and BGC823 cell lines. This compound reduced the production of lactate and inhibited the proliferation of gastric cancer cells [125]. Similar in vitro effects have been noted in medulloblastoma cell lines [126]. The in vitro anti-tumor effect has also been proven in cervical, liver, and non-small cell lung cancer cell lines [127,128,129]. A major problem in in vivo research is the poor ability of oxamate to penetrate the cell membrane. To achieve the intended effect in vitro, high concentrations of oxamate should be used in in vitro tests. These concentrations are difficult to achieve in in vivo experiments [130]. 

The mechanism of action of the next group of LDHA inhibitors is based on competing with NADH. Gossypol is a polyphenolic compound isolated from cotton seed that has shown the ability to inhibit the LDHA [131]. This compound inhibits the redox reactions catalyzed by NADH+/NADH-based enzymes such as LDHA and it is regarded as a non-selective inhibitor of this enzyme. Gossypol has shown anti-tumor activity based on various mechanisms of action, including suppression of anti-apoptotic proteins from the Bcl-2 family, cell cycle arrest, autophagy, and LDHA inhibition [132,133]. Anti-cancer properties of this compound have been proven in in vitro studies in such cancers as melanoma, breast cancer, cervical cancer, prostate cancer, colon cancer, leukemia, and glioma [134,135,136,137,138]. Promising results in in vivo studies with gossypol were obtained in the case of mouse xenografts of the BRW line obtained from a patient with primitive neuroectodermal tumor and mouse xenografts of two human head and neck squamous cell carcinoma cell lines [135,139]. Despite numerous clinical trials, gossypol has not been approved by the FDA for the treatment of any cancer. This is due to the non-specific toxicity of the compound related to the highly reactive chemical structure. As a result, gossypol can affect various cellular components, disrupting ion transport, macromolecule synthesis, calcium homeostasis, and processes related to the energy metabolism of the cell. The main side effects are hypokalemia, arrhythmias, renal failure, and muscle weakness. Therefore, gossypol analogs with lower biological reactivity are still being sought [137,140,141]. Another compound from the group of substances that inhibit LDHA through competition with NADH is the gossypol analog 3-dihydroxy6-methyl-7-(phenylmethyl)-4-propylnaphthalene-1-carboxylic acid (FX11, compound 7). It is referred to as a small-molecule, selective, competitive LDHA inhibitor [142]. The anti-tumor activity of FX11 has been observed in in vitro studies on gallbladder, prostate, and neuroblastoma cancer cells [143,144,145]. Research conducted by Le et al. proved that FX11 affects glycolysis-dependent cancer cell lines such as RCC4 (renal cell carcinoma), MCF-7 (breast carcinoma), and P493 (lymphoma B cells). Moreover, those studies showed that FX11 inhibits tumor growth in cancer xenograft models of human B-lymphoma and pancreatic cancer [124]. Despite the promising results in vitro and in vivo, FX11 did not prove to be a good candidate for the treatment of neoplastic diseases due to the high reactivity of the molecule, which may cause many side effects resulting from its biological activity [146]. The group of NADH-competitive LDHA inhibitors also includes quinoline-3-sulfonamides, which have shown anti-tumor activity in in vitro studies on hepatocellular carcinoma Snu398 cells. Unfortunately, due to the pharmacokinetic properties of these compounds related to incompatibility with oral bioavailability and low in vivo clearance, they exclude the use of quinoline-3-sulfonamides in vivo [147].

The mechanism of action of other LDHA inhibitors, N-hydroxyindoles, is based on the competition with pyruvate or NADH, which are natural substrates and cofactors of the reaction. N-hydroxyindoles show effective anti-proliferative activity in many cancer cell lines such as human pancreatic ductal adenocarcinoma, mesothelioma, ovarian cancer, and colorectal cancer. The inhibitory effect on cell proliferation was enhanced under hypoxic conditions [124,148,149]. Maftouh et al. [150] demonstrated the synergistic effect of N-hydroxyindole-NHI-1 with the chemotherapeutic agent gemcitabine in a pancreatic ductal adenocarcinoma cell line under hypoxic conditions [150]. An interesting direction is the use of N-hydroxyindole glycoconjugates in anti-cancer research. This strategy is based on a two-way targeting of tumor cells exhibiting the Warburg effect based on increased glucose uptake and high glycolysis. The use of a conjugate of a compound having a cytotoxic effect on cancer cells with glucose or another sugar allows for its increased uptake by a cancer cell, which is associated with increased expression of GLUT1 and a high rate of glycolysis. As a result, the therapy is directed mainly against cancer cells, sparing normal cells [151,152]. Further investigation of N-hydroxyindoles might be a promising direction in the treatment of neoplastic diseases.

Galloflavin is a molecule identified by Manerba et al. [153] as an inhibitor of both human isoforms of LDH. The mechanism of action is based on the preferential binding of the free enzyme without competition with the substrate or cofactor. Toxicity studies of galloflavin have shown that it does not cause lethal effects when it is administered at a maximum dose of 400 mg/kg [153]. In vitro studies demonstrated that galloflavin has an anti-tumor effect against various cancer cell lines such as breast cancer, endometrial cancer, Burkitt lymphoma, and hepatocellular carcinoma [154,155,156,157]. Wendt et al. [158] showed that galloflavin, in combination with metformin, has a strong anti-tumor effect against pancreatic ductal adenocarcinoma cells under both normoxic and hypoxic conditions. This can be the basis for the treatment of both solid tumors and disseminated metastases of this tumor [158].

### 3.2. Monocarboxylate Transporter 4

To maintain the intracellular pH of hemostasis, cells remove the lactate formed into the extracellular space using the lactate/H^+^ symporter and monocarboxylate transporter 4 (MCT4), which acidifies the microenvironment [159]. In many types of cancer, overexpression of monocarboxylate transporter 4 (MCT1), MCT4, or both is related to the metastatic capacity of neoplastic cells. For instance, Zhang et al. [160] found correlations between high MCT1 expression in tumor tissue samples taken from bladder cancer patients and lymph node metastases and distant metastases. Moreover, an in vitro study with bladder cancer cell lines with MCT1 knockdown showed an influence on the expression of proteins related to epithelial–mesenchymal transformation and inhibition of proliferation as well as migration and invasion of cancer cells [160]. The anti-cancer effect associated with the disruption of the functioning of MCT transporters has also been demonstrated in clear cell renal carcinoma and neuroendocrine prostate cancer [161,162]. One of the selective inhibitors of MCT1 is AZD3965, which has shown in vitro anti-tumor activity in diffuse large B cell lymphoma, non-Hodgkin’s lymphoma, and Burkitt lymphoma cell lines. Inhibition of MCT1 leads to lactate transport inhibition which causes an intracellular increase in lactate concentration. That was confirmed in in vivo studies on the Raji xenograft model (Burkitt lymphoma) [163]. AZD3965 showed the highest cytotoxic activity in cell lines characterized by a positive MCT1 profile and a negative MCT4 profile. Moreover, inhibition of proliferation and enhancement of the cytotoxic effect were observed when AZD3965 was combined with compounds such as GLS1 inhibitors, doxorubicin, and rituximab in the tested cell lines [163]. Currently, AZD3965 has been tested in phase I clinical trial in patients with solid tumors, diffuse large B cell lymphoma, and Burkitt lymphoma (NCT01791595). Another compound that has been identified as a potential MCT1 inhibitor is BAY-8002. Quanz et al. [164] showed that this compound significantly influences Daudi and Raji cells by inhibiting their proliferation. These studies have been confirmed in in vivo studies, where administration of BAY-8002 resulted in a reduction in a tumor mass. It is presumed that the increase in MCT2 and MCT4 expression is associated with increased resistance in cells chronically exposed to BAY-8002 [164].

### 3.3. Mitochondrial Pyruvate Carriers and Pyruvate Dehydrogenase

The resulting pyruvate may be oxidized in the TCA cycle. Pyruvate crosses the mitochondria via mitochondrial pyruvate carriers (MPCs). The MPC1 and MPC2 genes encode a multi-meric MPC complex that is located in the internal mitochondrial membrane [165,166]. The normal function of MPCs in cancer cells and the targeting of metabolism to mitochondrial respiration indicate that the Krebs cycle is functioning properly and the mitochondria do not need to be damaged, as argued by Warburg. Decreased MPC activity in some types of cancer can be used as a therapeutic target [165].

Another solution that regulates pyruvate metabolism is the regulation of the action of the pyruvate dehydrogenase complex (PDH) [167,168]. In the decarboxylation process, pyruvate is converted to acetyl-CoA, which enters the Krebs cycle. It is a reaction linking the glycolysis pathway with the citric acid cycle [167,168,169].

Targeting pytuvate dehydrogenase kinase (PDK) may sensitize cancer cells to chemotherapy and radiotherapy, or reduce drug resistance [32]. Dichloroacetate (DCA) inactivates PDK, leading to reactivation of PDH and resumption of metabolism from glycolysis to mitochondrial respiration. Coadministration of DCA and traditional chemotherapeutic agents in various tumor models have been studied extensively to produce a synergistic effect that would allow drug dose reductions and overcome drug resistance. Promising results were obtained with the combination of DCA and paclitaxel in non-small cell lung cancer, doxorubicin in hepatocellular carcinoma cells, and cisplatin in bladder cancer [170,171,172]. Much promising research has been conducted on combining DCA with other drugs that are mainly used in non-cancer diseases but also with several natural compounds [173]. Despite numerous in vivo studies and clinical trials with DCA in neoplastic diseases, this compound has not been approved by the FDA in the treatment of any type of cancer due to concerns about the safety and efficacy of this compound. The main factor limiting the use of DCA in therapy is the occurrence of dangerous side effects such as peripheral neuropathy. In addition, DCA is classified by the International Agency for Research on Cancer in the group of 2B compounds, i.e., possibly carcinogenic to humans [174].

## 4. The Pentose Phosphate Pathway

The pentose phosphate pathway (PPP), also known as the phosphogluconate pathway, is a metabolic pathway parallel to glycolysis and branches off in the first stage of glucose metabolism [175,176]. The PPP plays a role in the regulation of redox balance, being involved in the regulation of reactive oxygen species (ROS) levels, which promotes the proliferation of cancer cells [177]. Glycolysis and the pentose phosphate pathway run simultaneously, therefore the amount of oxidized glucose during cell respiration increases due to the common metabolites of both processes. The PPP is divided into two parts: irreversible oxidative and reversible non-oxidative [178].

Increased activity of the oxidative branch of the PPP occurs during multi-drug resistance in tumors. There is evidence that glucose-6-phosphate dehydrogenase (G6PD) may be a potential therapeutic target in the treatment of cancer. This is associated with the abundance of G6PD in many types of human cancer [179]. Several compounds are mentioned in the literature as inhibitors of G6PD. Dehydroepiandosterone (DHEA) is one of the potent, non-competitive inhibitors of G6PD, and its mechanism of action is to bind to the ternary enzyme–coenzyme–substrate complex. As a result of G6PD inhibition, the level of NADPH in the cell decreases, and the production of ROS dependent on this coenzyme is increased [180]. The use of DHEA in HeLa cervical cancer cells resulted in a reduced ability of cells to proliferate and migrate, as well as damage to the cytoskeleton of cells [181]. Other reports mention that inhibition of cell proliferation by DHEA may be due to a mechanism other than the inhibition of G6PD. It is suspected that the anti-proliferative effect might be related to changes in the expression of mitochondrial genes [182]. The main problem with the use of DHEA in anti-cancer therapy is rapid in vivo conversion into other hormones, which makes the drug inactive [183].

Another G6PD inhibitor is 6-aminonicotinamide (6-AN) which is an analog of nicotinamide. Inhibiting G6PD by 6-AN causes reduction in NADPH and accumulation of 6-phosphogluconate (6-PG) [184]. It is suspected that resistance to cisplatin in neoplastic cells is related to the action of the PPP. High expression and activity of G6PD have been verified in cisplatin-resistant ovarian cancer (C13, SKOV3/DDP), renal cancer (ccRCC), non-small cell lung cancer (A549/DDP), and bladder cancer (T24, TCCSUP) cell lines. The treatment of the abovementioned cancer cells with the combination of 6-AN as a G6PD inhibitor and cisplatin resulted in sensitization of the cells to cisplatin and reduced cell viability in comparison to cisplatin monotherapy [176,185,186,187,188]. In other conducted studies, Arbe et al. [189] showed that inhibition of G6PD by 6-AN sensitizes human melanoma cell lines to the cytotoxic effect of metformin which leads to apoptosis and necrosis of cells [189,190]. Natural compound polydatin (3,4′,5-trihydroxystilbene-3-β-d-glucoside; trans-resveratrol 3-β-mono-D-glucoside; piceid) is a glucoside of resveratrol. It inhibits the activity of G6PD activity which causes an increased level of cellular ROS and elevated endoplasmic reticulum stress. Mele et al. [179] proved that polydatin causes a strong inhibition of the proliferation of neoplastic cells and reduction in their invasiveness in vitro and in vivo in head and neck squamous carcinoma cells [179]. The anti-tumor effect of polydatin has also been investigated in nasopharyngeal carcinoma, hepatocellular carcinoma, lung cancer, and acute monocytic leukemia [191,192,193,194]. In the conducted phase II clinical trials, no major toxic effects on the main organs were found after taking polydatin for 3 months at a dose of 20-40 µg twice a day [195,196].

Zoledronic acid (ZA) is an FDA-approved drug that is used in the treatment of bone complications such as bone metastasis in multiple myeloma and other solid tumors and osteoporosis. ZA inhibits G6PD by decreasing its expression in bladder cancer cells which leads to reduced proliferation of cancer cells [197].

## 5. The Krebs Cycle

Reactions known as the tricarboxylic acid cycle, the citric acid cycle, or the Krebs cycle (TCA) are responsible for the oxidation of glucose and are the main metabolic pathway responsible for the energy supply to the cells, occurring in the mitochondrial matrix [198,199]. Cancer cells are characterized by metabolic and epigenetic changes in TCA cycle enzymes that correlate with disease progression. Therefore, some enzymes have become a potential therapeutic target, but a limitation is the high toxicity of the tested compounds [200,201].

The inhibitors of the TCA cycle include substances and factors such as zinc excess, toxic metals, vitamin B12 deficiency, and metformin. Among the many mechanisms of metformin’s action on cells, it is also pointed out that it can interfere with the TCA cycle and its metabolic intermediates, which are essential for the functioning of a cancer cell [202]. Metformin causes partial inhibition of NADH dehydrogenase, which disrupts the mitochondrial function, and in addition, in hepatocytes, it inhibits glycerol phosphate dehydrogenase. These processes disrupt oxidative phosphorylation and impair the effective transport of electrons from NADH and FADH2 through the electron transport chain [203,204]. Andrzejewski et al. [205], in studies conducted on isolated mitochondria, showed that metformin causes a general decrease in mitochondrial respiration and a compensatory increase in oxygen glycolysis. In addition, decreased glucose metabolism in the TCA cycle was noted [205].

Among the tested toxic metals, the influence on the TCA cycle and its activity is mentioned in the context of lead, cadmium, iron, manganese, chromium, and aluminum [206]. The use of toxic metal ions as anti-cancer drugs is impossible, however, further studies of the effect of metal ions on the pathways of energy metabolism may provide the basis for the identification of potential early biomarkers of an ongoing disease [207].

### 5.1. Glutamine and Fatty Acids as TCA Anaplerotic Substances

To maintain mitochondrial function with reduced availability of pyruvate, cancer cells supplement the metabolites of the TCA cycle in a process called anaplerosis. Such substances include glutamine and fatty acids [208,209,210]. In cancer patients, high levels of glutamine have been found compared to healthy people [210,211]. In the glutaminolysis reaction, glutamine is transformed into glutamate through glutaminase (GLS) [212]. 

**Figure 2:**
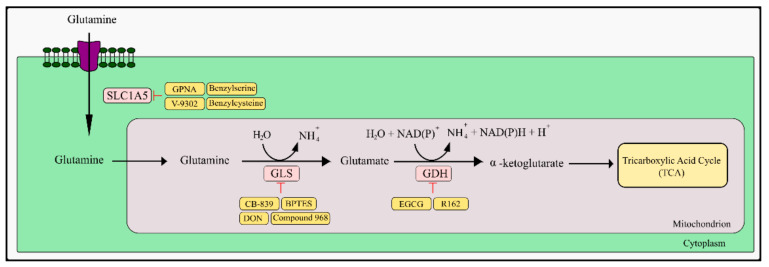
Schematic representation of glutamine transport, glutaminolysis pathway inhibitors, and its metabolic target. SLC1A5—glutamine transporter; GPNA—L-γ-glutamyl-p-nitroanilide; DON—6-diazo-5-oxo-L-norleucine; EGCG—epigallocatechin-3-gallate; GLS—glutaminase; GDH—glutamate dehydrogenase.

The next step is the conversion of glutamate to α-ketoglutarate (α-KG) by transamination of the amino group in the cytosol or mitochondria. This reaction can also take place during deoxidation catalyzed by glutamate dehydrogenase (GDH). There are several reports on GDH inhibitors in the literature, one of them is epigallocatechin-3-gallate, mentioned earlier in the context of PGAM1 inhibition. It has been shown that it allosterically inhibits GDH and inhibits the proliferation of glioma U251 cells in vitro and in the xenograft model [213]. However, the multi-directional action of EGCG makes it difficult to establish whether inhibition of the GDH enzyme is the primary cause of its anti-tumor activity in vivo. It also influences the enzymes that use NADPH as a reaction cofactor [214]. Another compound that has the ability to strongly inhibit GDH1 is the purpurin derivative R162. In vitro and in vivo studies have shown that this compound inhibits GDH more strongly and specifically in neoplastic cells than epigallocatechin-3-gallate. In addition, it does not affect other enzymes (Figure 2) [215].

The resulting α-KG becomes a substrate for further steps of the Krebs cycle, and also generates reducing equivalents to the electron transport chain (ETC), and the process of oxidative phosphorylation [209]. Research shows that cancer cells have an increased amount of glutamine transporters, including ASCT2 (SLC1A5). High levels of ASCT2 correlate with disease aggressiveness and reduced patient survival [216].

One of the inhibitors of the SLC1A5 transporter is L-γ-glutamyl-p-nitroanilide (GPNA). SLC1A5 is overexpressed in many primary human neoplasms which indicates its role in maintaining the necessary level of energy metabolism in cancer cells [217,218]. GPNA is a structural analog of glutamine and it inhibits Na^+^-dependent amino acid transporters such as SLC1A5. Attenuation of tumor cells as a result of limited L-glutamine uptake caused by GPNA inhibits signaling of rapamycin kinase complex 1 (mTORC1). In vitro and/or in vivo studies in which GPNA was used resulted in inhibition of the growth of cancer cells. This effect was noted in lung cancer, neuroblastoma, prostate cancer, multiple myeloma, breast cancer, and endometrial carcinoma [219,220,221,222,223,224,225]. Further studies are necessary to assess all possible mechanisms of action in both normal and cancer cells. In other performed studies, another potential inhibitor of SLC1A5, 2-amino-bis (aryloxybenzyl) aminobutanoic acid (V-9302), was identified. It is referred to as a small-molecule competitive inhibitor of the transmembrane glutamine flux. Its anti-tumor activity has also been demonstrated both in vitro and in vivo [226]. However, a new study performed by Bröer et al. [227] reports that in 143B osteosarcoma cells, HCC1806 breast cancer cells, and *Xenopus laevis* oocytes, V-9302 did not inhibit SLC1A5, but only SNAT2, SLC38A2, LAT1, and SLC7A5 [227]. Two other SLC1A5 inhibitors are benzylserine and benzylcysteine. They cause a competitive inhibition of the glutamine binding as a substrate to SLC1A5 [228]. Both of these compounds inhibit cancer cell growth: benzylserine in breast cancer and endometrial carcinoma, benzylcysteine in gastric cancer [225,229,230]. 

One of the best-studied glutaminase inhibitors is 6-diazo-5-oxo-L-norleucine (DON), which is a glutamine antagonist. The mechanism of action is based on the irreversible covalent bonding with the active site of the enzyme. It also inhibits another glutamine-dependent enzyme, glutamine amidotransferase [231,232]. Early literature reports indicate that DON causes cell growth inhibition of various types of cancer cells and has shown promising results in in vivo murine models of carcinomas, sarcomas, and leukemias [231,233,234]. Many conducted phase I and II clinical trials in the second half of the 20th century established unacceptable gastrointestinal toxicity (nausea, vomiting) when taking high doses of DON intermittently. This resulted in the limitation of the use of DON in clinical treatment regimens [231,235]. An excellent alternative to the administration of DON is the use of DON prodrugs, which allow minimizing the significant toxicity of DON and drug delivery to the appropriate target tissues. It has been suggested that central nervous system-developing neoplasms such as glioblastoma and medulloblastoma are good candidates to study the effect of DON prodrugs, due to the limited number of drugs with the possibility of crossing the blood–brain barrier, as well as other neoplasms characterized by a high dependence on glutamine [236]. JHU-083, Rais 5C, and Nedelcovych-13d can be mentioned among the synthesized prodrugs of DON. Studies performed by Hanaford et al. [237] have shown that JHU-083 induces growth inhibition of MYC-overexpressing medulloblastoma cell lines [237]. In vivo tests performed on a mouse model confirmed the in vitro tests [237,238,239].

Other GLS inhibitors include BPTES and its derivatives CB-839 and compound 968, which are allosteric inhibitors of GLS with different mechanisms of inhibition. BPTES has shown broad anti-tumor activity against such cancers as breast cancer, lymphoma, glioma, pancreatic cancer, non-small cell lung cancer, and kidney cancer [240,241,242,243,244,245]. Despite its high potential to inhibit the proliferation of neoplastic cells, BPTES is characterized by low water solubility and low bioavailability. Unfortunately, these features significantly limit the use of this compound in further clinical trials [246]. Searching for an effective and clinically applicable GLS inhibitor led Calither Bioscences to develop CB-839 (Telaglenastat^®^). CB-839 demonstrated anti-proliferative activity towards such cancers as breast, kidney, leukemia, and melanoma [247,248,249,250,251,252]. Currently, many clinical trials are being conducted with the status of “recruiting” and “active not recruiting” in various types of cancer with the use of CB-839 mainly in combination with other drugs used in anti-cancer therapy. Currently, FDA-approved CB-839 is being used in combination with cabozantinib for the treatment of patients with metastatic renal cell carcinoma who have received two or more prior lines of therapy based on the CANTATA trial (NCT03428217). In the case of another allosteric inhibitor of GLS, compound 968, there is a difference in the mechanism of action compared to bis-2-(5-phenylacetamido-1,3,4-thiadiazol-2-yl)ethyl sulfide (BPTES) and CB-839 in the form of binding to the monomeric interface of GLS, and not binding at the dimer interface [253]. This small-molecule inhibitor has shown anti-tumor activity in non-small cell lung cancer, glioblastoma, breast cancer, and ovarian cancer [254,255,256,257]. Additionally, in non-small cell lung cancer and ovarian cancer, compound 968 sensitizes tumor cells to the cytotoxic effect of erlotinib and paclitaxel, respectively [254,257].

### 5.2. Fatty Acids

Fatty acids are another type of anaplerotic substance that feeds TCAs. β-oxidation of fatty acids is a multi-stage catabolic process. During this process, mitochondrial conversion of long-chain fatty acids from the carboxyl terminus to acetyl-CoA takes place and the reduction of NAD to NADH and FAD to FADH_2_. The resulting acetyl-CoA is still oxidized in the mitochondria, while the resulting electrons are transferred to the respiratory chain, where energy in the form of ATP is produced [258,259,260,261].

**Figure 3:**
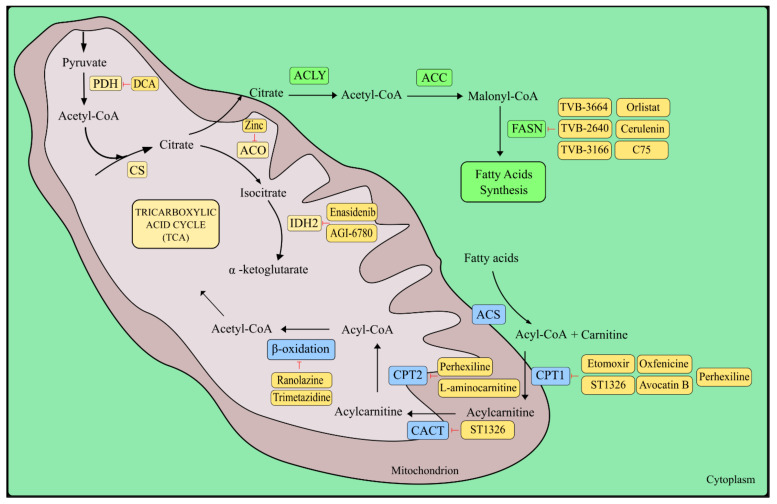
Graphical representation of pyruvate dehydrogenase complex (PDH), aconitase (ACO), isocitrate dehydrogenase 2 (IDH2), fatty acid synthesis, as well as metabolism inhibitors and their metabolic target. DCA—dichloroacetate; ACO—aconitase; ACLY—citrate lyase ATP; ACC—acetyl-CoA carboxylase; FASN—fatty acid synthase; ACS—acetyl-CoA synthetase; CPT1—carnitine palmitoyltransferase 1; CPT2—carnitine acyltransferase 2; CACT—carnitine-acylcarnitine translocase.

Treatment of neoplastic diseases based on the inhibition of β-oxidation of fatty acids may play a significant role in cancers that rely heavily on this pathway in the context of providing energy, e.g., prostate cancer [262]. The first interesting target in the context of anti-cancer therapy in the β-oxidation pathway of fatty acids is the enzyme carnitine palmitoyltransferase 1 (CPT1) located on the outer mitochondrial membrane, which converts fatty acyl-CoA to fatty acyl-carnitine [263,264,265]. One of the compounds showing a strong ability to irreversibly inhibit CPT1 (CPT1A and CPT1B), is glycidyl acid derivate etomoxir (ethyl-2-[6-(4-chlorophenoxy)hexyl] oxirane-2-carboxylate), which had been used in the treatment of type 2 diabetes and chronic heart failure. Due to the occurrence of hepatotoxicity, etomoxir is no longer used in the treatment of these diseases [266,267,268]. Research conducted by Schlaepfer et al. [262] demonstrated the anti-tumor activity of etomoxir in vitro in LNCaP, VCaP, and PC-3 prostate cancer cell lines. Etomoxir also caused a reduction in tumor growth in in vivo studies on the VCaP mouse xenograft model [262]. In studies performed on nasopharyngeal carcinoma, it was shown that the use of etomoxir causes sensitization of the cancer cells to radiotherapy, both in vitro and in vivo [269]. In addition, etomoxir has been shown to be cytotoxic in in vitro studies in such cancers as acute myeloid leukemia and breast cancer [270,271]. Another compound that selectively inhibits CPT1A is (R)-3-(3-tetradecylureido)-4-(trimethylammonio)butanoate (ST1326) [272,273]. In in vitro and in vivo studies performed by Pacelli et al. [274], ST1326 caused decreased viability of the Raji Burkitt lymphoma cell line and inhibited proliferation of cancer cells in xenograft models [274]. In addition, ST1326 demonstrated anti-tumor activity against several leukemia cell lines as well as cells obtained from patients with hematological malignancies [275,276]. Moreover, in AML studies, the synergistic effect of ST1326 with the inhibitor Bcl-2 ABT199 was noted [277]. Another CPT1 inhibitor with a limited number of scientific reports in the context of anti-cancer activity is oxfenicine (S-4-OH-phenyl-glycine) [278]. This compound showed anti-cancer activity in in vitro studies of malignant melanoma HBL cells [279]. The natural CPT1 inhibitor is avocatin B, a lipid derivative isolated from avocado fruit. Lee et al. [280] in conducted studies that showed that avocatin B decreases proliferation and reduces the viability of AML cells, inter alia, by inhibiting CPT1. Moreover, avocatin B does not affect normal stem hematopoietic cells (Figure 3) [280].

A compound that inhibits CPT1, but also CPT2, is perhexiline (2- (2, 2-dicyclohexylethyl) piperidine). Originally, perhexiline was an anti-angina drug and has been used since the 1970s [281,282]. Liu et al. [281] conducted research on chronic lymphocytic leukemia cells and discovered that perhexiline is highly effective in reducing the viability of these cancer cells in the stromal microenvironment. These results were confirmed in in vivo studies on a chronic lymphocytic leukemia transgenic mouse model [281]. 

The next step in the β-oxidation pathway is the transfer of fatty acyl-carnitine to the mitochondrial matrix via carnitine/acylcarnitine translocase (CACT), which is located on the inner membrane of the mitochondrion [283]. Pacilli et al. [274], in research conducted on the Raji Burkitt lymphoma cell line, proved that the ST1326 inhibitor causes inhibition not only of CPT1 but also of CACT. The effect is the inhibition of the proliferation of neoplastic cells, as well as the accumulation of lipids [274]. Reports on the occurrence of overexpression and increased activity of CACT in prostate cancer as well as abnormal regulation of CACT in bladder cancer contribute to the search for new compounds inhibiting this cotransporter [284,285].

The next step is the conversion of fatty acyl-carnitine to fatty acyl-CoA by carnitine acyltransferase 2 (CPT2) in the mitochondrial matrix. In addition to the previously mentioned perhexiline, the inhibitor of CPT2 is L-aminocarnitine [286]. However, there are no clear reports on the efficacy of this compound in the treatment of neoplastic diseases in in vitro and in vivo studies. To obtain the final product, i.e., acetyl-CoA, which enters the TCA cycle, acyl-CoA is cleaved by a repeating cycle of four reactions catalyzed by the enzymes [261]. Ranolazine and trimetazidine are considered to be 3-KAT inhibitors [287]. However, studies conducted by Ma et al. [288] proved that both of these compounds, when tested on cell lines, primary cells, and mice, did not inhibit fatty acid oxidation [288]. In the light of these reports, further in-depth research on inhibitors of the β-oxidation of fatty acids in cancer diseases is necessary, as it is a promising direction for further development of treatment strategies. 

Cancer cells can activate the de novo fatty acid synthesis pathway, which acts as energy reserves for the cancer cell. Not only inhibitors of the β-oxidation process but also inhibitors of de novo fatty acid synthesis are promising potential therapeutic targets in the treatment of cancer [289]. The enzymes involved in the synthesis of fatty acids are citrate ATP lyase (ACLY), acetyl-CoA carboxylase (ACC), and fatty acid synthase (FASN). Their expression often correlates with progression and prognosis and is used as a biomarker of metastases [290]. By carrying out the process of fatty acid synthesis, it is possible to supply lipids to rapidly proliferating cancer cells. The resulting fatty acids become a component of the cancer cell membrane, energy storage, and a substrate for the production of signaling molecules [291]. 

Many natural and synthetic compounds can inhibit FASN. The ones worth paying special attention to in the context of anti-cancer treatment are orlistat, cerulenin, C75, TVB-2640, TVB-3664, and TVB-3166. Orlistat is a reduced form of a natural product, lipostatin, and has been approved by the FDA for the treatment of obesity since 1999, as it inhibits pancreatic and gastric lipase. That inhibition blocks the absorption of free fatty acids in the gastrointestinal tract [292,293]. FASN inhibition by orlistat is based on irreversible binding to this enzyme and inhibition of the TE domain [294]. Orlistat has shown anti-tumor activity in preclinical studies, inter alia, in such cancers as ovarian, brain, head and neck, gastrointestinal, T-cell lymphoma, colorectal, prostate, melanoma, lung, pancreatic, and hepatocellular carcinoma [295,296,297,298,299,300,301,302,303,304,305]. Unfortunately, the limitation in conducting further clinical trials on orlistat is its poor solubility in water and poor absorption from the gastrointestinal tract [306].

Cerulenin is a non-competitive, specific, small-molecule FASN inhibitor and its mechanism of action is based on the reaction of its epoxide group with the FASN β-ketoacyl synthase domain [307]. It was one of the first natural FASN inhibitory compounds which showed anti-tumor activity in in vitro studies of breast cancer and ovarian cancer xenograft models [308,309]. Cerulenin has shown anti-tumor activity against such cancers as colon cancer, colorectal cancer, retinoblastoma, bladder cancer, and lung adenocarcinoma [310,311,312,313,314]. A problem in further studies on cerulenin is the high reactivity of the epoxy group of cysteine, which causes severe side effects. Based on the cerulenin mechanism of bonding to the enzyme, C75 was designed as a more chemically stable compound [315,316]. C75, compared to cerulenin, can interact with β-ketoacyl synthase, enoyl reductase, and thioesterase domains [317]. Despite the very promising results of C75 in in vitro and in vivo studies in many types of cancers, it shows side effects similar to cerulenin, including, among others, impact on food intake and severe weight loss [318,319,320].

TVB-2640 is a small-molecule, highly selective, oral FASN inhibitor. TVB-2640 was the first FASN inhibitor that entered advanced cancer clinical trials. Phase I trials included the treatment of patients with colon or other resectable cancers (NCT02980029). There are several phase II clinical trials, including treatment of patients with KRAS mutant non-small cell lung cancers with metastases (NCT03808558), a combination of TVB-2640, paclitaxel, and trastuzumab in treating patients with HER2-positive metastatic breast cancer (NCT03179904), and a combination of TVB-2640 with bevacizumab in patients with the first relapse of high-grade astrocytoma (NCT03032484) [321]. Two other novel FASN inhibitors, TVB-3664 and TVB-3166, show anti-tumor activity in many types of neoplasms in in vitro and in vivo tests, but, currently, there are no reports of their use in further clinical trials [322,323,324].

### 5.3. Isocitrate Dehydrogenase

Isocitrate dehydrogenase (IDH) catalyzes the oxidative decarboxylation reaction of isocitrate to α-ketoglutarate and CO_2_. The IDH1 and 2 mutations promote the formation of ROS, which induces oxidative stress leading to an increase in carcinogenicity [325,326,327]. Mutant IDH at positions R172 and R140, instead of catalyzing the conversion of isocitrate to alpha-ketoglutarate, catalyzes the conversion of alpha-ketoglutarate to beta-hydroxyglutarate (2-HG), the increase in which leads to hypermethylation of target genes and blockade of cell differentiation [328]. Among multiple types of cancers, IDH1 or/and IDH2 mutations have been identified, inter alia, in acute myeloid leukemia (AML), gliomas, chondrosarcoma, intrahepatic cholangiocarcinoma, thyroid carcinoma, and also rarely in prostate cancer, melanoma, and paraganglioma [329,330,331,332,333,334].

The best-known inhibitor of mutant IDH1 is ivosidenib (AG-120, Tibsovo^®^), which was approved by the FDA on 20 July 2018, for the treatment of adults with relapsed or refractory AML with a susceptible IDH1 mutation based on a clinical trial (NCT02074839). Ivosidenib is a small-molecule compound that is rapidly absorbed after oral administration. Studies have shown that administration of ivosidenib at a dose of 500 mg/day reduces total serum 2-HG levels by more than 90% [335,336]. Studies conducted by Popovici-Muller et al. [337] have shown that ivosidenib inhibits several IDH1-R132 mutants and had no inhibitory effect on WT or the IDH2 mutant [337]. On 2 May 2019, the FDA approved ivosidenib as monotherapy for newly diagnosed AML in adult patients with an IDH1 mutation not eligible for intensive chemotherapy [338]. Several different clinical trials are currently underway involving ivosidenib in combination with other anti-cancer drugs in AML, including, inter alia, a phase III clinical trial with azacytidine (NCT03173248), a phase I clinical trial with chemotherapeutics such as cytarabine and fludarabine (NCT04250051), and a phase I/II clinical trial with venetoclax with or without azacytidine (NCT03471260). Ivosidenib is also being investigated in a phase III clinical trial in previously treated advanced cholangiocarcinoma with IDH1 mutations (NCT02989857). Currently, 21 clinical trials in cancer diseases are being conducted with the use of ivosidenib with the status of not yet recruiting, recruiting, and active not recruiting (Supplementary Materials Table S1).

Other mutant IDH1 inhibitors include BAY1436032, olutasidenib, and IDH305. BAY1436032 is an allosteric oral inhibitor developed by Bayer IDH1 with the ability to pass through the blood–brain barrier [339]. Preclinical studies have shown the therapeutic efficacy of this compound in both AML and glioma models [340]. Unfortunately, a phase I study conducted to determine safety, tolerability, pharmacokinetics, pharmacodynamics, and preliminary clinical efficacy in patients with IDH1 mutation in advanced AML showed a low overall response rate even at the highest dose tested (NCT03127735). These results significantly limit the clinical development of BAY1436032 in AML [341]. Phase I clinical trials of BAY1436032 are ongoing in patients with advanced solid tumors, including glioma with the IDH1 mutation (NCT02746081). Promising results were shown by Chaturvedi et al. [342], who demonstrated a synergistic effect of BAY1436032 and azacitidine in two xenograft models of human AML with the IDH1 mutation [342]. 

Olutasidenib (FT-2102) is an orally administered mutant IDH1 inhibitor. Two clinical trials with olutasidenib are ongoing. The first is a phase I/II clinical trial in adult patients with relapsed/refractory or untreated AML and myelodysplastic syndrome. Olusidenib is being tested in this clinical trial as monotherapy or in combination with azacytidine (NCT02719574) [343]. A second ongoing phase I/II clinical trial in patients with advanced solid tumors and gliomas includes olutasidenib as a single agent and in combination with other anti-cancer drugs (NCT03684811). IDH-305 is currently in a phase I clinical trial in patients with advanced malignancies with IDH R132 mutation (NCT02381886). 

One of the best-studied and best-known mutant IDH2 inhibitors is enasidenib. It is an allosteric, small-molecule, selective, oral IDH2 inhibitor targeting mainly the R140Q and R172K IDH2 isoforms. Enasidenib inhibits the conversion of α-KG to 2-HG by binding to the allosteric site of the mutant enzyme, resulting in a significant reduction in serum 2-HG levels [344]. Based on a phase I/II clinical trial (NCT01915498), enasidenib (Idhifa^®^) was approved by the FDA on 1 August 2017, for the treatment of adult patients with relapsed or refractory AML with an IDH2 mutation [345]. This mutation must be detected using an FDA-approved test [338,346]. The ongoing clinical trials of enasidenib in cancer diseases are shown in the Supplementary Materials (Supplementary Materials Table S2). Another reported mutated IDH2 inhibitor is AGI-6780. It is an allosteric, selective compound that inhibits the IDH2 enzyme harboring the R140Q mutation. In vitro studies performed by Wang et al. showed that AGI-6780 induces differentiation of acute myelogenous leukemia and TF0-1 erythroleukemia cells [347]. Currently, there are no reports in the literature about the in vivo research conducted with the use of AGI-6780 in the treatment of neoplastic diseases.

## 6. Mitochondria as a Therapeutic Target in Cancer Treatment—Mitocans

Mitochondria are extremely valuable metabolic centers for cancer cells due to their higher metabolic needs and the necessity to maintain a relatively high level of ROS, which is related to the proliferation, migration, invasion, and metastasis of cancer cells [348]. Even though cancer cells are capable of oxidative glycolysis to obtain ATP, many types of cancers depend on OXPHOS, including the late stages of the disease [349,350,351]. Disruption of mitochondrial function through targeted treatment in cancer cells would disrupt the OXPHOS mechanism and other processes taking place in this organelle necessary for cancer cell survival. A group of compounds that disrupt the function of mitochondria are mitocans (from the combination of the words “mitochondria” and “cancer”) [352]. Due to the various mechanisms of action of mitocans, they have been divided into eight classes:

1. Class 1: hexokinase inhibitors.
2. Class 2: compounds targeting Bcl-2 family proteins.
3. Class 3: thiol redox inhibitors.
4. Class 4: VDAC/ANT-targeting drugs.
5. Class 5: electron transport chain-targeting drugs.
6. Class 6: lipophilic cations targeting inner membrane.
7. Class 7: TCA cycle-targeting drugs.
8. Class 8: mtDNA-targeting drugs [353].

Two classes of mitocans, hexokinase inhibitors and TCA-targeting drugs, have already been discussed above in this review. Although not all of groups of mitocans have a direct influence on the energy metabolism of the tumor cell, their use ultimately leads to the impairment of mitochondrial function and, as a result, death of the tumor cell. The mechanism of action of the second class of mitocans is related to the targeting of anti-apoptotic proteins from the Bcl-2 family (Bcl-2, Bcl-XL), which are overexpressed in cancer cells, thus preventing them from entering the apoptotic pathway [354,355]. The selective targeting of small-molecule compounds to the anti-apoptotic Bcl-2 proteins was mainly based on the development of BH3 mimetics. Their mechanism of action is based on binding to pro-survival proteins and thus sensitizing tumor cells to apoptosis [356]. Compounds belonging to this class include ABT-737, navitoclax (ABT-263), obatoclax (GX15-070), and venetoclax (ABT-199) [355]. Navitoclax is a derivative of ABT-737, and it is better tolerated and safer for patients, which has been proven in phase I studies in patients with non-small cell lung cancer (NSCLC) or lymphoid malignancies (NCT00445198, NCT00406809). The main side effect of this compound is dose-dependent thrombocytopenia, which makes it difficult to administer it on its own. However, there are reports of its high effectiveness in combination therapies, including erlotinib, gemcitabine, carboplatin, and paclitaxel in solid tumors [357,358,359]. The only compound targeting Bcl-2 family proteins that has been approved by the FDA for the treatment of cancerous diseases is venetoclax. It has been approved by the FDA for treatment of patients with 17p deleted relapsed/refractory chronic lymphocytic leukemia (CLL), a population with a very poor prognosis, and in combination with the hypomethylating agents azacitidine or decitabine or a low dose of cytarabine as a front-line therapy in AML patients who cannot tolerate aggressive chemotherapy [360,361]. Despite the high effectiveness of venetoclax in neoplastic diseases, the developing resistance of cancer cells is a major problem, which precludes long-term effective therapy [362]. 

The third and fourth classes of mitocans are associated with the redox balance in mitochondria of cancer cells. The third class of mitocans are compounds that inhibit the antioxidant system in cancer cells. Due to their already elevated ROS level, cancer cells are susceptible to the toxic effects of ROS overproduction [363]. Thiol redox inhibitors cause oxidation of the thiol groups or depletion of the mitochondrial GSH pool, which can lead to the death of cancer cells [363,364]. Examples of such compounds are phenethyl isothiocyanate (PEITC) and arsenic trioxide [365,366]. Research conducted by Trachtootam et al. [367] showed that the application of PEITC on CCL cells resulted in a significant increase in the level of ROS, caused by the depletion of the GSH pool, and, as a result, death of leukemia cells. Moreover, PEITC caused the death of fludarabine-resistant CLL cells [367]. The fourth class of mitocans are compounds such as 4-[N-(S-glutathionylacetyl)amino]phenylarsineoxide (GSAO), CD437, arsenic trioxide, and betulinic acid [353,368,369]. Their mechanism of action is based on targeting the VDAC/ANT1 permeability transition pore complex, which allows various molecules to pass through the mitochondrial membrane. These molecules include metabolites, ROS, apoptogenic factors, and small ions but also small molecules such as ADP and ATP [370]. Studies have shown that cancer cells exhibit a significantly increased expression of VDAC1 [371,372,373]. GSAO, through its inhibitory effect on ANT function, induced oxidative stress and apoptosis selectively in proliferating angiogenic endothelial cells. Due to this mechanism, GSAO can be considered an anti-angiogenic compound that prevents the development of blood supply in the tumor [369]. CD437 is a synthetic analog of all-trans retinoic acid and its action induces mitochondrial permeability transition pores through interaction with ANT. Studies performed on myeloma cells have shown that CD437 caused depolarization of the mitochondrial membrane associated with the formation of pores, which resulted in the induction of apoptosis in these neoplastic cells [374,375]. 

The fifth class of mitocans are electron transport chain inhibitors. Respiratory chain targeting offers the opportunity to develop new therapeutic strategies. The effects of compounds can focus on electron transport, proton gradient maintenance, electron to oxygen transfer, and ATP synthesis. OXPHOS inhibitors can be combined with immunological therapies to increase the chances of recovery [376,377]. Still, direct inhibitors of electron transport chain complexes are not used in the treatment of neoplastic diseases. It is associated in most cases with too low selectivity of compounds towards cancer cells. Therefore, it is necessary to search for compounds that disrupt the functioning of the mitochondrial electron transport chain and ATP synthase with a high affinity for cancer cells. Examples of the compounds with the ability to inhibit the complexes of the electron transport chain and ATP-synthase and their mechanisms of action in cancer cells are shown in the Supplementary Materials (Supplementary Materials Table S4).

The sixth class of mitocans are lipophilic cations targeting the inner membrane. Their mechanism of action is based on the fact that cancer cells have a much higher negative transmembrane potential than normal cells. As a result, lipophilic cations are more selective for neoplastic cells. Positively charged lipophilic cations penetrate the hydrophobic barriers of the cytoplasm and mitochondrial membranes and accumulate on the inner mitochondrial membrane [378]. This causes a disturbance in the functioning of the mitochondria, including processes involving ROS, cytochrome c, and pro-apoptotic factors, which leads to apoptosis and death of the neoplastic cell [379]. Compounds in this class of mitocans are rhodamine-123, F16, (KLAKKLAK)_2_, and triphenylphosphonium (TPP+)-based compounds. Rhodamine-123 showed a selective cytotoxic effect on cancer cells in studies carried out in the 1980s, which increased the interest of scientists in compounds from the group of delocalized lipophilic cationic agents [380,381]. Compound F16 also selectively accumulates on the inner mitochondrial membrane and was shown to be cytotoxic in an in vitro study performed by Wang et al. [382] on gastric (SGC-7901) and breast (MCF-7) cancer cell lines [382]. An interesting compound from this class of mitocans is a proapoptotic peptide (KLAKKLAK)_2_ conjugated with the cell-penetrating peptide, penetratin. This conjugate showed anti-proliferative activity in vitro at a concentration of 10 µM on such tumors as non-small cell lung carcinoma (A549, Epo40, Epo480), neuroblastoma (SK-N-SH), glioblastoma (U87, U251), and colon carcinoma (SW480) [383]. 

One of the strategies for the treatment of neoplastic diseases based on the use of delocalized lipophilic cations assumes the use of TPP+ moiety as a carrier of various molecules to the mitochondria of cancer cells. Studies using TPP+ as a carrier of compounds for mitochondria in the treatment of cancer include TPP+ conjugates with antioxidants, natural products, already known drugs, and enzyme inhibitors [368]. An example of a TPP-linked antioxidant with anti-cancer properties is MitoQ and its analog SkQ1. MitoQ showed anti-cancer activity in in vitro studies on the MDA-MB-231 breast cancer cell line. The compound caused cell cycle arrest in the G1/S phase and induction of autophagy and apoptosis in breast cancer cells [384]. In vitro and in vivo studies performed by Tania Capeloa’s research team have shown that MitoQ prevents breast cancer cell migration, invasion, formation of metastasis, and local recurrence in mice after surgery [385,386]. SkQ1 in fibrosarcoma (HT1080) and rhabdomyosarcoma (RD) cell lines causes inhibition of the proliferation of these cancer cells. In addition, in in vivo studies with a rhabdomyosarcoma xenograft nude mouse model, SkQ1 causes downregulation of the weight of subcutaneous tumors [368,387]. Further examples of TPP+ conjugates with antioxidants are Mito-CP and Mito-ChM and its acetate (Mito-ChMAc). Mito CP has anti-tumor activity against colon cancer cells and also inhibits medullary thyroid carcinoma in vitro and in vivo in xenograft models [368,388,389,390]. Mito-ChM and Mito-CHMAc showed an anti-proliferative effect in several breast cancer cell lines [368,391]. Mito-curcumin and Mito-resveratrol are examples of conjugates of TPP+ with natural products. Mito-curcumin has shown strong anti-tumor activity in in vitro studies using breast cancer (MCF-7, MDA-MB-231), neuroblastoma (SK-N-SH), prostate cancer (DU-145), and cervical cancer (HeLa) cell lines [392,393]. Research carried out with the use of Mito-resveratrol on colon cancer cells (CT-26) revealed its strong anti-cancer activity [394]. A promising group of natural compounds that have been tested in the context of TPP+ conjugates are natural triterpenic acids. It has been proved that betulin conjugate with triphenylphosphonium salts has better anti-tumor activity in comparison to betulin [395]. These conjugates showed an anti-proliferative effect against vinblastine-resistant breast cancer cells (MCF-7/Vinb), breast cancer cells (MCF-7), and prostate cancer cells (PC-3) [395,396]. Other triterpenic acids, betulinic and ursolic acid, in conjugates with TPP+ revealed anti-cancer activity against breast cancer cells (MCF-7) and neuroblastoma cells (TET21N) [395,397]. Moreover, triphenylphosphonium cations of betulinic acid derivatives have anti-tumor activity against the mastocytoma cell line (P-815) and murine Ehrlich ascites carcinoma [398]. Research performed by Jin et al. [399] showed that conjugate of glycyrrhetinic acid with TPP+ (2f) causes apoptotic death in lung cancer cells (A549) and has greater selectivity towards cancer cells compared to a single compound [399]. 

Another compound from the group of lipophilic cations that can be used as a carrier of natural compounds to the mitochondria of cancer cells is F16. Recently, two studies carried out by Dubinin et al. with the use of botulin-F16 and betulinic acid-F16 conjugates showed the toxic effect of conjugates on the functioning of the rat liver mitochondrion by, among others, inhibiting complex I of the respiratory chain and stimulating the production of hydrogen peroxide [400,401]. Moreover, the F6 conjugate has a protonophore-like effect and causes the permeabilization of the mitochondrial membrane [401]. The team of R. Csuk was involved in the synthesis and research of conjugates of rhodamine B and rhodamine 101 with triterpenoic acids [402,403,404,405]. They showed that many conjugates of rhodamine B with triterpenoic acids have a cytotoxic effect on cancer cells at nanomolar concentrations. The conjugates inhibited cell proliferation of such tumors as melanoma (518A2), colorectal adenocarcinoma (HT29), lung adenocarcinoma (A549), thyroid carcinoma (8505C), and others [395,405].

The use of TPP+ conjugates with drugs already used in treatment, such as chlorambucil, doxorubicin, or metformin, provides great hope in the development of this branch of treatment of neoplastic diseases. The use of conjugates may result in a significant increase in selectivity toward cancer cells, which will allow for a reduction in drug doses and a significant reduction in the severe side effects associated with the therapy [368,395]. Chlorambucil is a DNA-alkylating agent, and its main anti-cancer mechanism of action is DNA alkylation [406]. It is used in the treatment of many cancers, for example, chronic lymphocytic leukemia (CLL), Waldenström’s macroglobulinemia, and lymphomas such as Hodgkin’s disease and non-Hodgkin lymphoma [407,408]. Research performed by Milliard et al. [409] has shown that conjugate of TPP+ and chlorambucil targets and accumulates in mitochondria and, moreover, it causes mtDNA damage. Breast and pancreatic cancer cell lines used in this in vitro study were insensitive to the parent drug. Mito-chlorambucil caused the death of tumor cells and had a much stronger anti-cancer effect than the pure compound. Moreover, an in vivo study in a mouse xenograft model of human pancreatic cancer showed that Mito-chlorambucil slows down the development of the tumor [368,409,410]. Another drug conjugated with TPP+ is doxorubicin, widely used in the treatment of many neoplastic diseases. In vitro studies with wild-type and doxorubicin-resistant breast cancer cells (MDA-MB-435/WT, MDA-MB-435/DX) showed that Mito-doxorubicin has a strong cytotoxic effect on doxorubicin-resistant cancer cells in comparison to free doxorubicin. This demonstrates the possibility of using conjugated doxorubicin to reverse the drug resistance of cancer cells [411]. Moreover, studies performed on the conjugate of TPP+, doxorubicin, and hyaluronic acid revealed that it has a better anti-tumor effect than free doxorubicin in in vitro and in vivo research with MCF-7/ADR breast cancer cells [412]. Mitochondrially targeted metformin conjugated with TPP+ also demonstrated a significant anti-proliferation effect on pancreatic cancer cells in vitro and in vivo [413,414]. Attempts were made to test compounds that are TPP+ and enzyme inhibitor conjugates. In vitro studies carried out by Pathak et al. [415] on prostate cancer cell lines (PC3, DU145, LNCaP) revealed that Mito-DCA is much more cytotoxic to cancer cells than regular DCA. Moreover, Mito-DCA is characterized by greater selectivity in relation to neoplastic cells than DCA, because it does not adversely affect the energy metabolism of healthy cells. Mito-DCA caused mitochondrial dysfunction of tumor cells, disturbance of glycolysis, and apoptotic death of prostate cancer cells [368,415]. 

The eighth class of mitocans are compounds that target mitochondrial DNA (mtDNA). They may be related to targeting the cancer cell energy metabolism, because mtDNA encodes several subunits of complexes of the electron transport chain, necessary for OXPHOS. The mechanisms of action of this class of mitocans are based on the disruption of the stability of mtDNA and inhibition of mitochondrial DNA polymerase-γ. Vitamin K3 (menadione) in research conducted by Sasaki et al. [416] demonstrated the ability to inhibit the polymerase-γ of mtDNA. Moreover, it showed a cytotoxic effect on various cancer cell lines such as colon (HCT116, HCT115, SW620), lung (H1299, A549), breast (MCF-7), liver (HepG2), pancreas (PANC-1,) prostate (LNCaP, PC-3, DU145), B cell lymphoma (Raji), and cervical (HeLa) [416]. Other compounds that belong to the eighth class of mitocans are 1-methyl-4-phenyl-pyridinium (MPP+) and rhodocyanine dye MKT-077. MPP+ destabilizes the D-loop in mtDNA and MKT-077 induces mtDNA damage. They showed anti-proliferative activity on cervical cancer HeLa S3 cells and colon carcinoma CX-l cells [417,418]. 

## 7. Conclusions

Cancer was recognized as an altered metabolism disease 100 years ago, but in the last decade, many discoveries have been made towards reprogramming the energy metabolism of cancer cells, which is highly dynamic and heterogeneous. Metabolic changes in the tumor cell play a critical role in cancer features such as migration, invasion, and metastasis.

Due to fundamental metabolic differences between normal and cancer cells, targeting altered cellular metabolism is also recognized as a potential way to achieve therapeutic selectivity. Targeting certain points in the metabolic pathways can reduce the proliferation of cancer cells. In addition, after extensive research, checkpoint inhibitor therapy has emerged as the first-line therapy in many types of cancer. A huge problem in the searching for selective therapy is the plasticity of cancer cells and their hybrid phenotype. Metabolic changes, such as increased aerobic glycolysis, promotion of anaplerotic responses, and especially the dependence of cells on glutamine and fatty acid metabolism have become the subject of study. Research development must understand the relationship between metabolic pathways, redox regulation, and the role of mitochondria in metabolic processes, yet many compounds with favorable pharmacokinetics and safety profiles have been identified.

We think that using a combination of drugs aimed at cytoplasmic glycolysis and mitochondrial metabolism at the same time may be a promising anti-cancer strategy compared to monotherapy. The use of multiple inhibitors should disturb the various metabolic compartments within the tumor. Combination therapies targeting both glycolysis and OXPHOS may be more effective, but care must be taken not to make such therapies more toxic. Currently, the focus should be on conducting clinical trials involving the study of prognostic biomarkers aimed at metabolism concerning several pathways at the same time.

Knowledge about cancer metabolism is constantly evolving. More research and understanding of these changes are needed to develop new cancer therapy strategies and new drugs.

## Data Availability

Not applicable.

## Supplementary Materials

The following supporting information can be downloaded at: www.mdpi.com/article/10.3390/ijms23105572/s1.
