# Supplementary material for: Targeting Energy Metabolism in Cancer Treatment
Source: Int J Mol Sci. 2022 May 16;23(10):5572. doi: 10.3390/ijms23105572 (PMC9146201; doi:10.3390/ijms23105572)
Supplement: Supplementary file 1 [file ijms-23-05572-s001.zip › ijms-1683938-supplementary.pdf]

Table S1. Clinical trials of ivosidenib in cancer diseases with the status of not yet recruiting, recruiting, and active not recruiting.

NCT Number	Title	Phase	Status	Disease	Intervensions
NCT03564821	A Phase 1 Study of IDH1 Inhibition Using Ivosidenib as Maintenance Therapy for IDH1-mutant Myeloid Neoplasms Following Allogeneic Stem Cell Transplantation	1	Active, not recruiting	Myeloid Neoplasms with IDH1 mutation undergoing hematopoietic stem cell transplantation	Ivosidenib
NCT04955938	A Phase Ib Study of Fedratinib With IDH Inhibition in Advanced-Phase, IDH-Mutated Ph-Negative Myeloproliferative Neoplasms	1	Recruiting	IDH-Mutated Ph-Negative Myeloproliferative Neoplasms	Fedratinib plus Ivosidenib, Fedratinib plus Enasidenib
NCT04056910	Phase II Study of IDH1 Inhibitor Ivosidenib and Nivolumab in IDH1 Mutant Gliomas and Advanced Solid Tumors	2	Recruiting	Advanced solid tumors (nonresectable or metastatic) or enhancing gliomas with IDH1 mutation	Ivosidenib plus Nivolumab
NCT02074839	A Phase I, Multicenter, Open-Label, Dose-Escalation and Expansion, Safety, Pharmacokinetic, Pharmacodynamic, and Clinical Activity Study of Orally Administered AG-120 in Subjects With Advanced Hematologic Malignancies With an IDH1 Mutation	1	Recruiting	R/R Acute Myeloid Leukemia, Untreated Acute Myeloid Leukemia, Hematologic Malignancie, Myelodysplastic Syndromes with IDH1 mutation	Ivosidenib
NCT04195555	NCI-COG Pediatric MATCH (Molecular Analysis for Therapy Choice) - Phase 2 Subprotocol of AG-120 (Ivosidenib) in Patients With Tumors Harboring IDH1 Mutations	2	Recruiting	Advanced solid tumors, lymphoma, histiocytic disorders with IDH1 mutation	Ivosidenib
NCT03471260	Phase Ib/II Investigator Initiated Study of the IDH1-Mutant Inhibitor Ivosidenib (AG120) With the BCL2 Inhibitor Venetoclax +/- Azacitidine in IDH1-Mutated Hematologic Malignancies	1b/2	Recruiting	Acute Myeloid Leukemia, R/R Acute Myeloid Leukemia, Hematopoietic and Lymphoid System Neoplasm, Myelodysplastic Syndrome, Myeloproliferative Neoplasm with IDH1 mutation	Ivosidenib plus Azacitidine plus Venetoclax

NCT02073994	A Phase 1, Multicenter, Open-Label, Dose-Escalation and Expansion, Safety, Pharmacokinetic, Pharmacodynamic, and Clinical Activity Study of Orally Administered AG-120 in Subjects With Advanced Solid Tumors, Including Glioma, With an IDH1 Mutation	1	Active, not recruiting	Cholangiocarcinoma Chondrosarcoma Glioma Other Advanced Solid Tumors with IDH1 mutation	Ivosidenib
NCT04493164	Phase II Investigator Sponsored Study of CPX-351 in Combination With Ivosidenib for Patients With IDH1 Mutated Acute Myeloid Leukemia or High-Risk MDS	2	Recruiting	Acute myeloid leukemia, R/ R Acute Myeloid Leukemia, Myelodysplastic Syndrome, Myeloproliferative Neoplasm with IDH1 mutation	Ivosidenib plus Liposome-encapsulated Daunorubicin-Cytarabine (CPX-351)
NCT04176393	A Phase 1, Multicenter, Single-Arm Study Evaluating Pharmacokinetic, Pharmacodynamic, Safety, and Clinical Efficacy of Orally Administered Ivosidenib in Chinese Subjects With Relapsed or Refractory Acute Myeloid Leukemia With an IDH1 Mutation	1	Active, not recruiting	R/R Acute Myeloid Leukemia with IDH1 mutation	Ivosidenib
NCT04250051	Phase 1 Trial of Ivosidenib and FLAG Chemotherapy in Relapsed/Refractory IDH1+ Acute Myeloid Leukemia (AML)	1	Not yet recruiting	R/R Acute Myeloid Leukemia, R/R Myelodysplastic Syndrome, R Myeloproliferative Neoplasm with IDH1 mutation	Ivosidenib
NCT04774393	Phase 1b/2 Study of Oral Decitabine/Cedazuridine (ASTX727) and Venetoclax in Combination With the Targeted Mutant IDH1 Inhibitor Ivosidenib or the Targeted Mutant IDH2 Inhibitor Enasidenib	1b/2	Recruiting	R/R Acute Myeloid Leukemia with IDH1 mutation	Decitabine/cedazuridine (ASTX727) plus Venetoclax plus Ivosidenib or Enasidenib
NCT05010772	Oral Decitabine-Based Maintenance Therapy in Patients With AML in Remission	1	Recruiting	Acute Myeloid Leukemia	Decitabine plus Enasidenib or Gilteritinib or Ivosidenib or Venetoclax
NCT02677922	A Phase 1b/2 Open-label, Randomized Study of 2 Combinations of Isocitrate	1b/2	Active, not recruiting	Newly diagnosed Acute Myeloid Leukemia with IDH1 or IDH2 Mutation	Oral Ivosidenib plus Subcutaneous

	Dehydrogenase (IDH) Mutant Targeted Therapies Plus Azacitidine: Oral AG-120 Plus Subcutaneous Azacitidine and Oral AG-221 Plus SC Azacitidine in Subjects With Newly Diagnosed Acute Myeloid Leukemia Harboring an IDH1 or an IDH2 Mutation, Respectively, Who Are Not Candidates to Receive Intensive Induction Chemotherapy				(SC) azacitidine, Oral Enasidenib plus Subcutaneous (SC) azacitidine
NCT04278781	Phase II Study of AG-120 in IDH1 Mutant Chondrosarcoma	2	Recruiting	Advanced/metastatic or recurrent Chondrosarcoma with IDH1 mutation	Ivosidenib
NCT05209074	A Phase I, Single-Center, Open-Label, Dose De-escalation and Expansion Study Ivosidenib + mFOLFIRINOX in Patients With Resectable Pancreatic Adenocarcinoma	1	Not yet recruiting	Pancreatic Ductal Adenocarcinoma	Ivosidenib plus mFOLFIRINOX
NCT02632708	A Phase 1, Multicenter, Open-Label, Safety Study of AG-120 or AG-221 in Combination With Induction Therapy and Consolidation Therapy in Patients With Newly Diagnosed Acute Myeloid Leukemia With an IDH1 and/or IDH2 Mutation	1	Active, not recruiting	Newly Diagnosed Acute Myeloid Leukemia With an IDH1 and/or IDH2 Mutation	Ivosidenib or Enasidenib with 2 types of AML induction therapies (cytarabine with either daunorubicin or idarubicin) and 2 types of AML consolidation therapies (mitoxantrone with etoposide or cytarabine).
NCT04088188	A Phase I, Multi-Center, Open Label, Dose De-Escalation and Expansion Study of Gemcitabine and Cisplatin With AG120 or Pemigatinib for Advanced Cholangiocarcinoma	1	Recruiting	Advanced Cholangiocarcinoma	Gemcytabine and Cisplatin plus Ivosidenib, Gemcytabine and Cisplatin plus Pemigatinib
NCT03173248	A Phase 3, Multicenter, Double-Blind, Randomized, Placebo-Controlled Study of AG-120 in Combination With Azacitidine in Subjects ≥ 18 Years of Age With Previously Untreated Acute Myeloid Leukemia With an IDH1 Mutation	3	Active, not recruiting	Newly Diagnosed Acute Myeloid Leukemia, Untreated Acute Myeloid Leukemia, Acute Myeloid Leukemia Arising From Myelodysplastic	Ivosidenib plus azacitidine

				Syndrome (MDS) with IDH1 mutation	
NCT03343197	A Phase 1, Multicenter, Randomized, Controlled, Open-Label, Perioperative Study of AG-120 and AG-881 in Subjects With Recurrent, Non-Enhancing, IDH1 Mutant, Low Grade Glioma	1	Active, not recruiting	Glioma with IDH1 mutation	Ivosidenib or AG-881
NCT03839771	A Phase 3, Multicenter, Double-blind, Randomized, Placebo-controlled Study of Ivosidenib or Enasidenib in Combination With Induction Therapy and Consolidation Therapy Followed by Maintenance Therapy in Patients With Newly Diagnosed Acute Myeloid Leukemia or Myelodysplastic Syndrome With Excess Blasts-2, With an IDH1 or IDH2 Mutation, Respectively, Eligible for Intensive Chemotherapy.	3	Recruiting	Newly Diagnosed Acute Myeloid Leukemia, Myelodysplastic Syndrome With Excess Blasts-2 with IDH1 mutation or IDH2 mutation	Ivosidenib plus chemotherapy, Enasidenib plus chemotherapy
NCT03503409	A Single-arm Phase II Multicenter Study of IDH1 (AG 120) Inhibitor in Patients With IDH1 Mutated Myelodysplastic Syndrome	2	Recruiting	Myelodysplastic Syndromes with IDH1 mutation	Ivosidenib

Table S2. Clinical trials of enasidenib in cancer diseases with the status of not yet recruiting, recruiting, and active not recruiting.

NCT Number	Title	Phase	Status	Disease	Intervensions
NCT01915498	A Phase 1/2, Multicenter, Open-Label, Dose-Escalation and Expansion, Safety, Pharmacokinetic, Pharmacodynamic, and Clinical Activity Study of Orally Administered AG-221 in Subjects With Advanced Hematologic Malignancies With an IDH2 Mutation	1/2	Active, not recruiting	Advanced Hematologic Malignancies With an IDH2 Mutation	Enasidenib
NCT04092179	Phase Ib/II Study of IDH2 Inhibitor Enasidenib in Combination With BCL2 Inhibitor Venetoclax in Patients With IDH2-Mutated Myeloid Malignancies (ENAVEN-AML)	1b/2	Recruiting	R/R Acute Myeloid Leukemia with IDH2 Mutation	Enasidenib plus Venetoclax
NCT04281498	A Phase II Open-label Study of Combined Ruxolitinib and Enasidenib in Patients With Accelerated/Blast-phase Myeloproliferative Neoplasm or Chronic-phase Myelofibrosis With an IDH2 Mutation	2	Recruiting	Accelerated/Blast-phase Myeloproliferative Neoplasm or Chronic-phase Myelofibrosis With an IDH2 Mutation	Ruxolitinib plus Enasidenib
NCT04955938	A Phase Ib Study of Fedratinib With IDH Inhibition in Advanced-Phase, IDH-Mutated Ph-Negative Myeloproliferative Neoplasms	1	Recruiting	IDH-Mutated Ph-Negative Myeloproliferative Neoplasms	Fedratinib plus Ivosidenib, Fedratinib plus Enasidenib
NCT03515512	A Phase I Study of IDH2 Inhibition Using Enasidenib as Maintenance Therapy for IDH2-mutant Myeloid Neoplasms Following Allogeneic Stem Cell Transplantation	1	Active, not recruiting	Acute Myeloid Leukemia, Chronic Myelomonocytic Leukemia with IDH2 mutation	Enasidenib
NCT04522895	IDH2-Post-Allo-Trial: Enasidenib as Consolidation or Salvage Therapy for Patients With IDH2 Mutated AML or MDS Following Allogeneic Blood Stem Cell Transplantation	2	Recruiting	Acute Myeloid Leukemia, Myelodysplastic Syndromes, Chronic Myelomonocytic Leukemia in remission after allo-SCT or relapsed after allo-SCT with IDH2 mutation	Enasidenib
NCT04203316	An Open-Label Feasibility Study to Assess the Safety and Pharmacokinetics of Enasidenib in Pediatric Patients With Relapsed/Refractory Acute	2	Recruiting	R/R Acute Myeloid Leukemia with IDH2 mutation	Enasidenib

	Myeloid Leukemia (R/R-AML) With an Isocitrate Dehydrogenase-2 (IDH2) Mutation				
NCT03720366	A Phase 1, 2-Part, Multicenter, Open-Label, 3-Arm Study to Determine the Effect of Enasidenib (CC-90007) on the Pharmacokinetics of Single Oral Doses of Caffeine, Dextromethorphan, Fexofenadine, Flurbiprofen, Midazolam, Omeprazole, Pioglitazone, and Rosuvastatin in Patients With Acute Myeloid Leukemia	1	Active, not recruiting	Acute Myeloid Leukemia with IDH2 mutation	Enasidenib plus Arm 1 probe or Arm 2 probe or Arm 3 probe
NCT03728335	Pilot Trial of Enasidenib (AG-221) Maintenance Post Allogeneic Hematopoietic Cell Transplantation in Patients With IDH2 Mutation	1	Recruiting	Acute Myeloid Leukemia with IDH2 mutation following donor stem cell transplant	Enasidenib
NCT04774393	Phase 1b/2 Study of Oral Decitabine/Cedazuridine (ASTX727) and Venetoclax in Combination With the Targeted Mutant IDH1 Inhibitor Ivosidenib or the Targeted Mutant IDH2 Inhibitor Enasidenib	1b/2	Recruiting	R/R Acute Myeloid Leukemia with IDH1 mutation	Decitabine/cedazuridine (ASTX727) plus Venetoclax plus Ivosidenib or Enasidenib
NCT02577406	A Phase 3, Multicenter, Open-label, Randomized Study Comparing the Efficacy and Safety of AG-221 (CC-90007) Versus Conventional Care Regimens in Older Subjects With Late Stage Acute Myeloid Leukemia Harboring an Isocitrate Dehydrogenase 2 Mutation	3	Active, not recruiting	R/R Acute Myeloid Leukemia with IDH2 mutation	Enasidenib
NCT05010772	Oral Decitabine-Based Maintenance Therapy in Patients With AML in Remission	1	Recruiting	Acute Myeloid Leukemia	Decitabine plus Enasidenib or Gilteritinib or Ivosidenib or Venetoclax
NCT02677922	A Phase 1b/2 Open-label, Randomized Study of 2 Combinations of Isocitrate Dehydrogenase (IDH) Mutant Targeted Therapies Plus Azacitidine: Oral AG-120 Plus Subcutaneous Azacitidine and Oral AG-221 Plus SC Azacitidine in Subjects With Newly Diagnosed Acute Myeloid Leukemia Harboring an IDH1 or an IDH2	1b/2	Active, not recruiting	Newly diagnosed Acute Myeloid Leukemia with IDH1 or IDH2 Mutation	Oral Ivosidenib plus Subcutaneous (SC) azacitidine, Oral Enasidenib plus Subcutaneous (SC) azacitidine

	Mutation, Respectively, Who Are Not Candidates to Receive Intensive Induction Chemotherapy				
NCT02632708	A Phase 1, Multicenter, Open-Label, Safety Study of AG-120 or AG-221 in Combination With Induction Therapy and Consolidation Therapy in Patients With Newly Diagnosed Acute Myeloid Leukemia With an IDH1 and/or IDH2 Mutation	1	Active, not recruiting	Newly Diagnosed Acute Myeloid Leukemia With an IDH1 and/or IDH2 Mutation	Ivosidenib or Enasidenib with 2 types of AML induction therapies (cytarabine with either daunorubicin or idarubicin) and 2 types of AML consolidation therapies (mitoxantrone with etoposide or cytarabine).
NCT05282459	A Phase Ib/II, Single Center, Open-Label, Safety and Efficacy Study to Improve Anemia in Subjects on Enasidenib With Lower Risk Myelodysplastic Syndrome and Non-proliferative Chronic Myelomonocytic Leukemia Without an IDH2 Mutation	1b/2	Recruiting	Myelodysplastic Syndrome and Non-proliferative Chronic Myelomonocytic Leukemia Without an IDH2 Mutation	Enasidenib mesylat
NCT03383575	Targeted Therapy With the IDH2-Inhibitor Enasidenib (AG221) for High-Risk IDH2-Mutant Myelodysplastic Syndrome	2	Active, not recruiting	IDH2-mutant Myelodysplastic Syndrome	Enasidenib in combination with azacitidine
NCT03744390	A Single-arm Phase II Multicenter Study of IDH2 (AG-221) Inhibitor in Patients With IDH2 Mutated Myelodysplastic Syndrome	2	Recruiting	Myelodysplastic Syndrome, Acute myeloblastic Leukemia with IDH 2 mutation	Enasidenib
NCT03839771	A Phase 3, Multicenter, Double-blind, Randomized, Placebo-controlled Study of Ivosidenib or Enasidenib in Combination With Induction Therapy and Consolidation Therapy Followed by Maintenance Therapy in Patients With Newly Diagnosed Acute Myeloid Leukemia or Myelodysplastic Syndrome With Excess Blasts-2, With an IDH1 or IDH2 Mutation, Respectively, Eligible for Intensive Chemotherapy	3	Recruiting	Newly Diagnosed Acute Myeloid Leukemia, Myelodysplastic Syndrome With Excess Blasts-2 with IDH1 or IDH2 mutation	Ivosidenib plus chemotherapy, Enasidenib plus chemotherpy

Table S3. Selected *in vitro* and *in vivo* research of inhibitors in cancer cell energy metabolic pathway.

Enzyme/Transporter	Inhibitor	Type of research	Type of cancer	References
**Glycolysis and glucose transporters**				
GLUT1	WZB117	*In vitro*	non–small cell lung cancer (A549, H1299)<br>breast cancer (MCF7)	Liu et al. 2012 [1]
		In vivo	xenograft model of non–small cell lung cancer (A549)	
GLUT2	Polyphenolic Floretin	*In vitro*	breast cancer (MDA-MB-231)	Wu et al. 2018 [2]
			colon cancer (COLO205)	Lin et al. 2016 [3]
GLUT4	Ritonavir	*In vitro*	multiple myeloma (L363, JJN3, KMS11)	Dalva-Aydemir et al. 2015 [4]
		In vivo	xenograft model of multiple myeloma (KMS11-GFP)	
HK2	2-DG	*In vitro*	ovarian cancer (SKOV3, IGROV1, IGROV-R10)<br>mesothelioma (MSTO-211H, NCI-H28)<br>glioblastoma (U251, GLI15)<br>head and neck squamous cancer (SCC12B2, SQ2OB, SCC61)<br>liver cancer (HepG2)<br>colon cancer (HT29D4)	Zhang et al. 2006 [5]
	3-BP	*In vitro*	malignant peripheral nerve sheath tumor (S462, NSF1, T265)	Linke et al. 2020 [6]
			mesothelioma (NCI-H28)	Philippe et al. 2012 [7]
			nasopharyngeal carcinoma (HNE1, CNE-2Z)	Zou et al. 2015 [8]
	BNBZ	*In vitro*	pancreatic cancer (SW1990)	Zheng et al. 2021 [9]
		In vivo	xenograft models of<br>pancreatic cancer (SW1990)<br>colon adenocarcinoma (SW480)	

ALDOA	UM0112176	*In vitro*	non-small cell lung cancer (KLN205, hNSCLC)<br>pancreatic adenocarcinoma (BxPC3)	Gizak et al. 2019 [10]
	Bisphosphonate-based inhibitor (Compound 2)	*In vitro*	cervical cancer (Hela)	Heron et al. 2018 [11]
GAPDH	KA	*In vitro*	melanoma (SK-MEL28, UACC-62, SK-MEL2, M14, SK-MEL5)<br>breast cancer (BT-474)<br>lung cancer (NCI-H522)<br>multiple myeloma (RPMI -8226)	Liberti et al. 2017 [12]
		In vivo	orthotopic breast cancer model (BT-474)	
	DC-1563	*In vitro*	breast cancer (BT-549, MCF-7, MDA-MB-231)<br>colon cancer (HCT116)<br>lung cancer (A549)	Li et al. 2020 [13]
PGK1	NG52	*In vitro*	glioma (U87, U251)	Wang et al. 2021 [14]
		In vivo	glioma patient-derived xenografts model	
PGAM1	PGMI-004A	*In vitro*	non-small cell lung carcinoma (H1299)<br>acute myelogenous leukemia (KG-1a)<br>head and neck squamous cell carcinoma (LN212)<br>myelogenous leukemia (K562)<br>erthroleukemia (HEL)<br>eosinophilic leukemia (EOL1)<br>acute monocytic leukemia (Molm14)	Hitosungi et al. 2012 [15]
		In vivo	xenograft model (H1299)	
	HKB99	*In vitro*	non-small cell lung cancer (PC9, HCC827, H1975, A549, HCC827ER-erlotynib resistant cells)	Huang et al. 2019 [16]

		In vivo	xenograft model (PC9, HCC827, HCC827ER)	
	MJE2	*In vitro*	breast cancer (MDA-MB-231)	Evans et al. 2005 [17]
	EGCG	*In vitro*	non-small cell lung carcinoma (H1299)<br>breast cancer (MDA-MB-231)	Li et al. 2017 [18]
	Xanthone derivatives	*In vitro*	non-small cell lung carcinoma (H1299)<br>breast cancer (MCF-7)<br>pancreatic cancer (PANC-1)	Wang et al. 2018 [19]
	KH3	*In vitro*	pancreatic cancer (SW1990, PANC-1, AsPC-1, MIA PaCa-2)	Wen et al. 2019 [20]
		In vivo	orthotopic mouse model of pancreatic ductal adenocarcinoma (MIA PaCa-2)	
			patient-derived xenograft models of pancreatic ductal adenocarcinoma	
ENO2	Phosphonoacetohydroxamate	*In vitro*	glioblastoma (D-423- cells with ENO1 deletion)	Muller et al. 2012 [21]
	POMHEX	*In vitro*	glioblastoma (D-423- cells with ENO1 deletion)	Lin et al. 2020 [22]
	SF2312	*In vitro*	glioblastoma (D-423- cells with ENO1 deletion)	Leonard et al. 2016 [23]
PKM2	Metformin	*In vitro*	osteosarcoma (HOS; metformin increases sensitivity of cells to cisplatin)	Shang et al. 2017 [24]
			bladder cancer (T24, UMUC3; metformin increases sensitivity of cells to pirarubicin)	Su et al. 2018 [25]
	Benserazide	*In vitro*	melanoma (SK-MEL5, SK-MEL28)	Zhou et al. 2020 [26]
		In vivo	xenograft model of melanoma (SK-MEL5, SK-MEL28)	
	Shikonin	*In vitro*	bladder cancer (T24, RT112; shikonin potentiates the responses of cells to cisplatin)	Wang et al. 2017 [27]
		In vivo	syngeneic mouse model of bladder cancer (MB49)	

		In vitro	cholangiocarcinoma (KKU-055, KKU-100, KKU-213A)	Thonsri et al. 2020 [28]
		In vitro	esophageal cancer (EC109, EC9706)	Tang et al. 2018 [29]
		In vivo	mice model of esophageal cancer (EC109)	
	Resveratrol	*In vitro*	cervical cancer (Hela)<br>colon cancer (DLD1)<br>breast cancer (MCF-7)	Wu et al. 2016 [30]
			melanoma (MV3, A375)	Zhao et al. 2018 [31]
	Vitamin K	*In vitro*	cervical cancer (Hela)	Chen et al. 2012 [32]
	Gliotoxin	*In vitro*	glioblastoma (U87,U251)<br>acute promyelocytic leukemia (HL-60)<br>chronic myelogenous leukemia (K-562)<br>breast cancer (MCF-7)<br>non-small cell lung cancer (H1975)<br>prostate cancer (PC-3)<br>colon cancer (HCT116)<br>cervical cancer (Hela)	Tang et al. 2020 [33]
	Compound 3k	*In vitro*	colon cancer (HCT116)<br>cervical cancer (Hela)<br>lung cancer (H1299)	Ning et al. 2017 [34]
**Pyruvate and it`s further fate**				
LDHA	Oxamate	*In vitro*	gastric cancer (SGC7901, BGC823)	Zhao et al. 2015 [35]
			medulloblastoma (UW402, Res256)	Valvona et al. 2018 [36]
			cervical cancer (SW756, C33A)	Stone et al. 2019 [37]
			hepatocarcinoma (Dt81Hepa1-6)	Cassim et al. 2018 [38]
			non-small cell lung cancer (H1299, A549)	Qiao et al. 2021 [39]
	Gossypol	*In vitro*	melanoma (SK-MEL19, SK-MEL28)<br>leukemia (K562)	Shelley et al. 1999 [40]

			cervical cancer (Sihas)<br>lung cancer (H69)	
			glioma (U373, U138, U87, HS683)	Coyle et al. 1994 [41]
			breast cancer (MCF-7)	Gilbert et al. 1995 [42]
			prostate cancer (PC-3, LNCaP, DU-145)	Volate et al. 2010 [43]
			colon cancer (HCT116, DLD-1)	Lan et al. 2015 [44]
		In vitro	head and neck squamous carcinoma (UM-SCC-17B, UM-SCC-1)	Wolter et al. 2006 [45]
		In vivo	orthotopic xenograft model of head and neck squamous carcinoma (UM-SCC-17B, UM-SCC-1)	
	FX11	*In vitro*	prostate cancer (PC-3, DU-145)	Xian et al. 2015 [46]
			gallbladder cancer (GBC-SD, NOZ, SGC-996)	He et al. 2018 [47]
			neuroblastoma (SK-N-AS, SK-N-SH, IMR-32, LAN-1, BE(2)-C)	Rellinger et al. 2017 [48]
		In vitro	renal carcinoma (RCC4)<br>breast cancer (MCF-7)<br>B-lymphoma (P493)	Le et al. 2010 [49]
		In vivo	xenograft model of human B-lymphoma (P493) and pancreatic cancer (P198)	
	Quinoline-3-sulfonamides	*In vitro*	hepatocellular carcinoma (SNU398)	Billiard et al. 2013 [50]
	NHI-1	*In vitro*	pancreatic cancer (PANC-1, MIA PaCa-2)	Granchi et al. 2011 (a) [51]
			ovarian cancer (ADDP, A2780/cOHP)<br>colorectal cancer (H630)<br>mesothelioma (NIH-H2452, NIH-H2052, MSTO-211H, NIH-H28)	Granchi et al. 2011 (b) [52]
	Galloflavin	*In vitro*	endometrial cancer (ECC-1, Ishikawa cells)	Han et al. 2015 [53]

			Burkitt lymphoma (Raji, Daudi, Louckes)	Vettraino et al. 2013 [54]
			breast cancer (MCF-7, MCF-Tam, MDA-MB-231)	Farabegoli et al. 2012 [55]
			liver cancer (PLC/PRF/5)	Manerba et al. 2017 [56]
MCT1	AZD3965	*In vitro*	Burkitt lymphoma (Raji)<br>Non Hodgkin's lymphoma (SU-DHL-10)<br>Diffuse large B cell lymphoma (WSU-DLCL-2)	Curtis et al. 2017 [57]
		In vivo	xenograft model of Burkitt`s lymphoma (Raji)	
	BAY-8002	*In vitro*	Burkitt`s lymphoma (Raji, Daudi)	Quanz et al. 2018 [58]
		In vivo	xenograft model of Burkitt`s lymphoma (Raji)	
PDK	DCA	*In vitro*	liver cancer (HepG2; sensitize cells to doxorubicin)	Korga et al. 2019 [59]
		In vitro	lung cancer (A549; resensetize sells to paclitaxel)	Sun et al. 2017 [60]
		In vitro	bladder cancer (HTB-9, HTB-5)	Woolbright et al. 2018 [61]
		In vivo	mouse xenograft model of bladder cancer (HTB-9)	
**The pentose phosphate pathway**				
G6PD	DHEA	*In vitro*	cervical cancer (Hela)	Fang et al. 2016 [62]
	6-AN	*In vitro*	ovarian cancer (C13)- overcomes resistance to cisplatin	Catanzaro et al. 2015 [63]
			ovarian cancer (SKOV3/DDP))- overcomes resistance to cisplatin	Xu et al. 2018 [64]
			renal cancer (ccRCC)- overcomes resistance to cisplatin	Lucarelli et al. 2015 [65]
			cell lung cancer (A549/DDP))- overcomes resistance to cisplatin	Hong et al. 2018 [66]

			bladder cancer (T24, TCCSUP)- overcomes resistance to cisplatin	Chen et al. 2018 [67]
			melanoma (A375, SB2, M8)- potentiated the cytotoxic effects of metformin	Arbe et al. 2020 [68]
	Polydatin	*In vitro*	nasopharyngeal carcinoma (CNE)<br>cervical cancer (HeLa)<br>hepatoma (SMMC-7721)<br>epidermal carcinoma (A431)	Liu et al. 2011 [69]
			acute monocytic leukemia (MOLT-4)	Wang et al. 2016 [70]
		In vitro	head and neck squamous carcinoma (UMSCC103)	Mele et al. 2018 [71]
		In vivo	orthotopic xenograft model of head and neck squamous carcinoma (UMSCC103)	
		In vitro	liver cancer (HepG2, SMMC-7721)	Jiao et al. 2018 [72]
		In vivo	xenograft model of liver cancer (HepG2)	
	ZA	*In vitro*	bladder cancer (T24)	Wang et al. 2015 [73]
**Glutamine as TCA anaplerotic substance**				
GLS	JHU-083	*In vitro*	medulloblastoma (D425MED)	Hanaford et al. 2019 [74]
		In vivo	xenograft model of medulloblastoma (D425MED)	
	BPTES	*In vitro*	glioblastoma (D54-R132H IDH1 mutation)	Seltzer et al. 2010 [75]
			lymphoblastoid cell line (P493)	Le et al. 2012 [76]
			pancreatic cancer (8988T)	Son et al. 2013 [77]
			breast cancer (MCF10A-NeuT, SK-BR-3, MDA-MB-453)	Qie et al. 2014 [78]
			non-small cell lung cancer (NCI-H647, NCI-H1703)	van den Heuvel et al. 2012 [79]
	CB-839	*In vitro*	breast cancer (HCC1806, MDA-MB-231)	Gross et al. 2014 [80]

			lung cancer (A549)	Ruan et al. 2019 [81]
			colon cancer (HCT116)	
			kidney cancer (Caki-1)	
			pancreatic cancer (SW1990)	
			acute myeloid leukemia (Molm14, OCI-AML3, MV4;11)	Matre et al. 2014 [82]
	Compound 968	*In vitro*	non-small cell lung cancer (HCC827ER, NCI-H1975)	Xie et al. 2015 [83]
			glioblastoma (GBM1, GBM10, GBM14)	Kahlert et al. 2016 [84]
			ovarian cancer (Hey, SKOV3, IGROV-1; sensitize cells to paclitaxel)	Yuan et al. 2016 [85]
GDH	EGCG	*In vitro*	glioma (U251)	Zhang et al. 2016 [86]
		In vivo	xenograft model of glioma (U251)	
	R162	*In vitro*	lung cancer (NCI-H1299, A549)<br>erythroleukemia (KG-1a, HEL)<br>acute monocytic leukemia (MOLM-14)<br>chronic myelogenous leukemia (K-562)<br>breast cancer (SKBR3, MDA-MB-231)	Jin et al. 2015 [87]
		In vivo	xenograft model of non-small cell lung carcinoma (NCI-H1299)	
SLC1A5 (ASCT2)	GPNA	*In vitro*	lung cancer (A549)	Hassanein et al. 2013 [88]
			neuroblastoma (BE-2C)	Ren et al. 2015 [89]
			prostate cancer (PC-3)	Wang et al. 2015 [90]
			breast cancer (HCC1806)	van Geldermalsen et al. 2016 [91]
			endometrial carcinoma (Ishikawa, HEC1A, KLE)	Marshall et al. 2017 [92]

	V-9302	*In vitro*	colon cancer (HCT116, COLO205, HT29, RKO)	Schulte et al. 2018 [93]
		In vivo	xenograft model of colon cancer (HCT116, HT29)	
	Benzylserine	*In vitro*	endometrial carcinoma (Ishikawa, HEC1A, KLE, RL95-2)	Marshall et al. 2017 [92]
			breast cancer (HCC1806, MDA-MB-231)	van Geldermalsen et al. 2018 [94]
	Benzylcysteine	*In vitro*	gastric cancer (SGC-7901)	Sun et al. 2013 [95]
**β-oxidation of fatty acids**				
CPT1	Etomoxir	*In vitro*	prostate cancer (LNCaP, VCaP, PC-3)	Schlaepfer et al. 2014 [96]
		In vivo	xenograft model of prostate cancer (VCaP)	
		In vitro	nasopharyngeal carcinoma (CNE2-IR, HK1-IR + radiotherapy)	Tan et al. 2018 [97]
		In vivo	xenograft model of nasopharyngeal carcinoma (CNE2-IR)	
		In vitro	acute myeloid leukemia (OCI-AML3, MOLM13)	Samudio et al. 2010 [98]
			breast cancer (MCF-7, ZR751)	Jariwala et al. 2021 [99]
	ST1326	*In vitro*	Burkitt`s lymphoma (Raji)	Pacilli et al. 2013 [100]
		In vivo	xenograft model of Burkitt`s lymphoma (Raji)	
		In vitro	acute myeloid leukemia (U937, MOLM13, OCIAML2, HL60, HL60/MX2, KG1) acute lymphoid leukemia (CEM S, CEM R, CCFR-CEM, RAJI, MOLT4, RS4;11, JURKAT)	Ricciardi et al. 2015 [101]
	Oxyfenicine	*In vitro*	melanoma (HBL)	Mascagna et al. 1992 [102]
	Avocatin B	*In vitro*	acute myeloid leukemia (OCI-AML2)	Lee et al. 2015 [103]

CPT1/CPT2	Perhexiline	*In vitro*	chronic lymphocytic leukemia cell obtained from patients	Liu et al. 2016 [104]
		In vivo	chronic lymphocytic leukemia transgenic mouse model	
**Fatty acids synthesis**				
FASN	Orlistat	*In vitro*	head and neck squamous cell carcinoma (rSCC-61)	Mims et al. 2015 [105]
			glioma (LN229, LT68, MW21)	Grube et al. 2014 [106]
			colorectal cancer (HT-29)	Czumaj et al. 2019 [107]
			prostate cancer (LNCaP, PC-3)	Chuang et al. 2019 [108]
			pancreatic cancer (PANC-1)	Sokolowska et al. 2017 [109]
			hepatocellular carcinoma (Hep3B)	You et al. 2019 [110]
			gastrointestinal stromal tumor (GIST48, GIST430)	Li et al. 2017 [111]
		In vitro	T cell lymphoma (Dalton's lymphoma cells)	Kant et al. 2012 [112]
		In vivo	murine model of a transplantable T cell lymphoma of spontaneous origin, designated as Dalton's lymphoma (DL)	
		In vitro	melanoma (B16-F10 murine melanoma cells)	Carvalho et al. 2008 [113]
		In vivo	melanoma model for spontaneous metastasis (B16-F10 murine melanoma cells)	
		In vitro	non-small cell lung cancer (H1299, A549, LCC)	Zhou et al. 2021 [113]
		In vivo	mouse xenograft model of non-small cell lung cancer (LCC)	
		In vivo	cisplatin-resistant ovarian tumour xenograft model in mice (A2780cis)	Papaevangelou et al. 2018 [114]
	Cerulenin	*In vitro*	breast cancer (ZR-75-1, SKBR3, MCF-7)	Pizer et al. 1996 [115]

		colon cancer (HT29, LoVo)	Chang et al. 2016 [116]
		retinoblastoma (Y79)	Deepa et al. 2011 [117]
		lung adenocarcinoma (A549, H1299)	Gouw et al. 2017 [118]
		bladder cancer (253J, 5637)	Jiang et al. 2012 [119]
	In vivo	xenograft model of drug-resistant ovarian cancer (OVCAR-3)	Pizer et al. 1996 [120]
	In vitro	colorectal cancer (HCT116, RKO)	Shiragami et al. 2013 [121]
	In vivo	xenograft model of colorectal cancer (HCT116)	
C75	*In vitro*	prostate cancer (LNCaP, PC-3)	Rae et al. 2020 [122]
	In vitro	hepatocellular carcinoma (HepG2, SMMC7721)	Gao et al. 2006 [123]
TVB-2640	*Clinical trial*	Phase 1 Study. Pharmacodynamic Effects of Fatty Acid Synthase (FASN) Inhibition With TVB-2640 in Resectable Colon Cancer and Other Resectable Cancers; a Window Trial.	NCT02980029
	Clinical trial	A Phase 2 Multi-center Pharmacodynamics Study of TVB-2640 in KRAS Mutant Non-small Cell Lung Carcinomas	NCT03808558
	Clinical trial	Phase 2 Trial to Evaluate the Efficacy of the FASN Inhibitor, TVB-2640, in Combination With Trastuzumab Plus Paclitaxel or Endocrine Therapy in Patients With HER2+ Metastatic Breast Cancer Resistant to Trastuzumab-Based Therapy	NCT03179904
	Clinical trial	A Phase 2 Investigator Initiated Study to Determine the Efficacy and Safety of TVB-2640 in Combination With Bevacizumab in Patients With First Relapse of High Grade Astrocytoma	NCT03032484

	TVB-3664	*In vitro*	colorectal cancer (Caco2, HT29)	Zaytseva et al. 2018 [124]
		In vivo	patient-derived xenograft model of colorectal cancer	
	TVB-3166	*In vitro*	lung cancer (CALU-6, NCI-H82, CALU-3, A427, NCI-H23, A549, NCI-H2347, NCI-H2122)<br>colon cancer (T84, LS-1034, HCT116, SNU-C1, COLO205, NCI-H508, HT29, HT55)<br>breast cancer (MDA-MB-231, MDA-MB-468, BT549, MCF7)<br>ovarian cancer (OVCAR 5, CaOV4)<br>prostate cancer (22RV1)<br>Burkitt`s lymphoma (Raji, Daudi)	Ventura et al. 2015 [125]
		In vivo	xenograft model of pancreatic cancer (PANC-1)<br>xenograft model of ovarian cancer (OVCAR-8)<br>patient-derived xenograft model of non-small-cell lung cancer	
		In vitro	bladder cancer (T24, UMUC3)	Tao et al. 2019 [126]

GLUT1: glucose transporter 1; GLUT2: glucose transporter 2; GLUT4: glucose transporter 4; HK2: hexokinase type 2; 2-DG: 2-deoxyglucose; 3-BP: 3-bromopyruvate; BNBZ: benitrobenrazide; ALDOA: aldolase A; GAPDH: glyceraldehyde 3-phosphate dehydrogenase; KA: koningic acid; MG: methylglyoxal; PGK1: phosphoglycerate kinase 1; PGAM1: phosphoglycerate mutase 1; EGCG: epigallocatechin-3-gallate; ENO2: γ-enolase; PK: pyruvate kinase; LDHA: lactate dehydrogenase isoform A; NHI-1: N-hydroxyindole, MCT1: monocarboxylate transporter 1; PDK: pyruvate dehydrogenase kinase; DCA: dichloroacetate; G6PD: glucose-6-phosphate dehydrogenase; DHEA: dehydroepiandosterone; 6-AN: 6-aminonicotinamide; ZA: zoledronic acid; GLS: glutaminase; GDH: glutamate dehydrogenase; SLC1A5: glutamine transporter; GPNA: L-γ-glutamyl-p-nitroanilide; CPT1: carnitine palmitoyltransferase 1; CPT2: carnitine acyltransferase 2; FASN: fatty acid synthase

Table S4. Examples of the inhibitors of the mitochondrial electron transport chain and ATP-synthase and their mechanisms of action in cancer cells.

Electron transport chain	Inhibiors	Mechanism of action	Cancer	Reference
Complex I	Metformin	Metformin reversibly inhibits mitochondrial complex I and reduces hypoxic activation of HIF-1α. Metformin causes inhibition of cancer cell proliferation in the presence of high glucose concentration in growth media and cancer cell death in conditions of low glucose availability.	lung cancer colorectal cancer	Wheaton et al. 2014 [127]
	IM156	IM156 inhibits mitochondrial complex I, which decreases NADH oxidation and activation of AMPK. IM156 indicates decrease in oxygen consumption rate.	lymphoma	Izreig et al. 2020 [128]
	Canagliflozin	Inhibition of proliferation of cancer cells is associated with inhibition of complex I, which causes reduction of ATP level and activation of AMPK.	lung cancer, prostate cancer	Villani et al. 2016 [129]
	BAY 87-2243	BAY 87-2243 causes induction of cancer cell death *in vitro* and reduction of tumor growth *in vivo* associated with complex I inhibition, which leads to oxygen consumption rate reduction and high level of ROS. In cancer cells maintained in the low glucose medium compound caused ATP depletion and induction of cancer cell death.	melanoma	Schöckel et al. 2015 [130]
	JCI-20679	JCI-20696 induces cancer cells growth inhibition mediated by the mitochondrial complex I inhibition.	panel of 39 cancer cell lines	Akatsuka et al. 2016 [131]
	Celastrol	Celastrol initiates apoptotic and necrotic cancer cell death through complex I inhibition which leads to ROS accumulation. Elevated level of ROS causes cell cycle arrest, inhibition of HSP90 function and JNK activation.	lung cancer, liver cancer	Chen et al. 2011 [132]
	AG311	AG311 causes complex I inhibition, HIF-1α stabilization, mitochondrial membrane depolarization, inhibition of tyrosine kinases and thymidylate synthase which leads to antitumor and antimetastatic effect on cancer cells.	breast cancer	Bastian et al. 2017 [133]
	Xanthohumol	Xanthohumol induces apoptosis of cancer cells through complex I inhibition, which leads to elevation of ROS level.	lung cancer, cervical cancer	Zhang et al. 2015 [134]

	Kalkitoxin	Kalkitoxin inhibits complex I, which causes decrease of oxygen consumption in cancer cells. This leads to inhibition of HIF-signaling and apoptosis of cancer cells. Moreover kalkitoxin targets tumor angiogenesis.	breast cancer	Morgan et al. 2015 [135]
	IACS-010759	Compound causes complex I inhibition, reduction of the activity of HIF-1 pathway and cancer cell apoptosis.	acute myeloid leukemia, ENO1 mutated glioblastoma	Molina et al. 2018 [136]
	CAI	Carboxyamidotriazole (CAI) inhibits complex I and causeses cancer cells growth reduction.	lung cancer	Stephenson et al. 2020 [137]
	Mubritinib	Mubritinib causes complex I inhibition.	acute myeloid leukemia	Baccelli et al. 2019 [138]
	MIBG	Meta-iodobenzylguanidine (MIBG) inhibits complex I and III of electron transport chain, causes reduction of the production of ATP and cancer cells death.	acute lymphoblastic leukemia	Cornelissen et al. 1995 [139]
	Verrucosidin	Verrucosidin causes complex I inhibition and induces cancer cell death in the hypoglycemic conditions.	breast cancer	Thomas et al. 2013 [140]
Complex II	α-Tocopheryl	α-Tocopheryl (α-TOS) inhibits succinate dehydrogenase (SDH) activity of complex II which, causes accumulation of ROS and cancer cell apoptosis.	breast cancer	Dong et al. 2008 [141]
	MitoVES	MitoVES causes inhibition of electron transfer from complex II to complex III, ROS accumulation and apoptosis of cancer cells.	acute T cell leukemia	Dong et al. 2011 [142]
	Atpenin A5 derivative	Atpenin A5 derivative inhibits complex II and leads to cancer cells death in both normoxic and hypoxic conditions.	prostate cancer	Wang et al. 2017 [143]
	Troglitazone	Inhibition of complex II by troglitazone causes massive apoptotic cancer cell death.	acute T cell leukemia	Soller et al. 2007 [144]

	DT-010	DT-010 inhibits complex II which leads to accumulation of ROS, loss of mitochondrial membrane potential, low ATP production and apoptosis of cancer cells.	breast cancer	Wang et al. 2016 [145]
Complex III	Atovaquone	Atovaquone targets the CoQ10-dependence of mitochondrial complex III which causes induction of oxidative stress, decreased oxygen consumption rate and death of cancer cells.	breast cancer	Fiorillo et al. 2016 [146]
	MIBG	MIBG inhibits complex I and III of electron transport chain, causes reduction of the production of ATP and cancer cells death.	acute lymphoblastic leukemia	Cornelissen et al. 1995 [139]
	Mahinine	Mahinine inhibits complex III of electron transport chain causes accumulation of ROS, induction of DNA damage response, Chk1/Chk2 upregulation and G0/G1 arrest of cancer cells.	glioblastoma	Bhattacharya et al. 2014 [147]
	Azoxystrobin	Azoxystrobin induces inhibition of complex III which leads to elevated level of ROS, loss of mitochondrial membrane potential and apoptosis of cancer cells.	oral cancer	Chen et al. 2020 [148]
Complex IV	Arsenic trioxide	Arsenic trioxide causes inhibition of complex IV which leads to accumulation of ROS, depolarization of mitochondrial membrane, decrease of ATP production and cancer cell death.	breast cancer	Sun et al. 2011 [149]
	Mitotane	Mitotane inhibits the activity of complex IV of electron transport chain which leads to cancer cell death.	adrenocortical carcinoma	Hescot et al. 2013 [150]
ATP-synthase	GBOXIN	Inhibition of ATP-synthase and death of cancer cells.	glioblastoma	Shi et al. 2019 [151]
	Angiostatin	Inhibition of ATP-synthase by the binding to α/β subunits.	colon cancer	Jung et al. 2007 [152]

References

1. Liu, Y.; Cao, Y.; Zhang, W.; Bergmeier, S.; Qian, Y.; Akbar, H.; Colvin, R.; Ding, J.; Tong, L.; Wu, S.; et al. A Small-Molecule Inhibitor of Glucose Transporter 1 Downregulates Glycolysis, Induces Cell-Cycle Arrest, and Inhibits Cancer Cell Growth *In Vitro* and *In Vivo*. *Mol Cancer Ther* **2012**, *11*, 1672–1682, doi:10.1158/1535-7163.MCT-12-0131.
2. Wu, K.-H.; Ho, C.-T.; Chen, Z.-F.; Chen, L.-C.; Whang-Peng, J.; Lin, T.-N.; Ho, Y.-S. The Apple Polyphenol Phloretin Inhibits Breast Cancer Cell Migration and Proliferation via Inhibition of Signals by Type 2 Glucose Transporter. *Journal of Food and Drug Analysis* **2018**, *26*, 221–231, doi:10.1016/j.jfda.2017.03.009.
3. Lin, S.-T.; Tu, S.-H.; Yang, P.-S.; Hsu, S.-P.; Lee, W.-H.; Ho, C.-T.; Wu, C.-H.; Lai, Y.-H.; Chen, M.-Y.; Chen, L.-C. Apple Polyphenol Phloretin Inhibits Colorectal Cancer Cell Growth via Inhibition of the Type 2 Glucose Transporter and Activation of P53-Mediated Signaling. *J. Agric. Food Chem.* **2016**, *64*, 6826–6837, doi:10.1021/acs.jafc.6b02861.
4. Dalva-Aydemir, S.; Bajpai, R.; Martinez, M.; Adekola, K.U.A.; Kandela, I.; Wei, C.; Singhal, S.; Koblinski, J.E.; Raje, N.S.; Rosen, S.T.; et al. Targeting the Metabolic Plasticity of Multiple Myeloma with FDA-Approved Ritonavir and Metformin. *Clin Cancer Res* **2015**, *21*, 1161–1171, doi:10.1158/1078-0432.CCR-14-1088.
5. Zhang, X.D.; Deslandes, E.; Villedieu, M.; Poulain, L.; Duval, M.; Gauduchon, P.; Schwartz, L.; Icard, P. Effect of 2-Deoxy-D-Glucose on Various Malignant Cell Lines In Vitro. *Anticancer Research* **2006**, *26*, 3561–3566.
6. Linke, C.; Wösle, M.; Harder, A. Anti-Cancer Agent 3-Bromopyruvate Reduces Growth of MPNST and Inhibits Metabolic Pathways in a Representative in-Vitro Model. *BMC Cancer* **2020**, *20*, 896, doi:10.1186/s12885-020-07397-w.
7. Philippe, I.; Xiao-Dong, Z.; Edwige, L.; Marie-Hélène, L.; Stéphane, A.; Hubert, L.; Laurent, P. Experimental Results Using 3-Bromopyruvate in Mesothelioma: In Vitro and in Vivo Studies. *J Bioenerg Biomembr* **2012**, *44*, 81–90, doi:10.1007/s10863-012-9415-6.
8. Zou, X.; Zhang, M.; Sun, Y.; Zhao, S.; Wei, Y.; Zhang, X.; Jiang, C.; Liu, H. Inhibitory Effects of 3-Bromopyruvate in Human Nasopharyngeal Carcinoma Cells. *Oncol Rep* **2015**, *34*, 1895–1904, doi:10.3892/or.2015.4147.
9. Zheng, M.; Wu, C.; Yang, K.; Yang, Y.; Liu, Y.; Gao, S.; Wang, Q.; Li, C.; Chen, L.; Li, H. Novel Selective Hexokinase 2 Inhibitor Benitrobenrazide Blocks Cancer Cells Growth by Targeting Glycolysis. *Pharmacological Research* **2021**, *164*, 105367, doi:10.1016/j.phrs.2020.105367.
10. Gizak, A.; Wiśniewski, J.; Heron, P.; Mamczur, P.; Sygusch, J.; Rakus, D. Targeting a Moonlighting Function of Aldolase Induces Apoptosis in Cancer Cells. *Cell Death Dis* **2019**, *10*, 712, doi:10.1038/s41419-019-1968-4.
11. Heron, P.W.; Abellán-Flos, M.; Salmon, L.; Sygusch, J. Bisphosphonate Inhibitors of Mammalian Glycolytic Aldolase. *J. Med. Chem.* **2018**, *61*, 10558–10572, doi:10.1021/acs.jmedchem.8b01000.
12. Liberti, M.V.; Dai, Z.; Wardell, S.E.; Baccile, J.A.; Liu, X.; Gao, X.; Baldi, R.; Mehrmohamadi, M.; Johnson, M.O.; Madhukar, N.S.; et al. A Predictive Model for Selective Targeting of the Warburg Effect through GAPDH Inhibition with a Natural Product. *Cell Metab* **2017**, *26*, 648-659.e8, doi:10.1016/j.cmet.2017.08.017.
13. Li, T.; Tan, X.; Yang, R.; Miao, Y.; Zhang, M.; Xi, Y.; Guo, R.; Zheng, M.; Li, B. Discovery of Novel Glyceraldehyde-3-Phosphate Dehydrogenase Inhibitor via Docking-Based Virtual Screening. *Bioorganic Chemistry* **2020**, *96*, 103620, doi:10.1016/j.bioorg.2020.103620.
14. Wang, W.; Jiang, Z.; Hu, C.; Chen, C.; Hu, Z.; Wang, A.; Wang, L.; Liu, J.; Wang, W.; Liu, Q. Pharmacologically Inhibiting Phosphoglycerate Kinase 1 for Glioma with NG52. *Acta Pharmacol Sin* **2021**, *42*, 633–640, doi:10.1038/s41401-020-0465-8.
15. Hitosugi, T.; Zhou, L.; Elf, S.; Fan, J.; Kang, H.-B.; Seo, J.H.; Shan, C.; Dai, Q.; Zhang, L.; Xie, J.; et al. Phosphoglycerate Mutase 1 Coordinates Glycolysis and Biosynthesis to Promote Tumor Growth. *Cancer Cell* **2012**, *22*, 585–600, doi:10.1016/j.ccr.2012.09.020.
16. Huang, K.; Liang, Q.; Zhou, Y.; Jiang, L.; Gu, W.; Luo, M.; Tang, Y.; Wang, Y.; Lu, W.; Huang, M.; et al. A Novel Allosteric Inhibitor of Phosphoglycerate Mutase 1 Suppresses Growth and Metastasis

of Non-Small-Cell Lung Cancer. *Cell Metabolism* **2019**, *30*, 1107-1119.e8, doi:10.1016/j.cmet.2019.09.014.

17. Evans, M.J.; Saghatelian, A.; Sorensen, E.J.; Cravatt, B.F. Target Discovery in Small-Molecule Cell-Based Screens by in Situ Proteome Reactivity Profiling. *Nat Biotechnol* **2005**, *23*, 1303–1307, doi:10.1038/nbt1149.
18. Li, X.; Tang, S.; Wang, Q.-Q.; Leung, E.L.-H.; Jin, H.; Huang, Y.; Liu, J.; Geng, M.; Huang, M.; Yuan, S.; et al. Identification of Epigallocatechin-3- Gallate as an Inhibitor of Phosphoglycerate Mutase 1. *Front. Pharmacol.* **2017**, *8*, 325, doi:10.3389/fphar.2017.00325.
19. Wang, P.; Jiang, L.; Cao, Y.; Zhang, X.; Chen, B.; Zhang, S.; Huang, K.; Ye, D.; Zhou, L. Xanthone Derivatives as Phosphoglycerate Mutase 1 Inhibitors: Design, Synthesis, and Biological Evaluation. *Bioorganic & Medicinal Chemistry* **2018**, *26*, 1961–1970, doi:10.1016/j.bmc.2018.02.044.
20. Wen, C.-L.; Huang, K.; Jiang, L.-L.; Lu, X.-X.; Dai, Y.-T.; Shi, M.-M.; Tang, X.-M.; Wang, Q.-B.; Zhang, X.-D.; Wang, P.-H.; et al. An Allosteric PGAM1 Inhibitor Effectively Suppresses Pancreatic Ductal Adenocarcinoma. *Proc. Natl. Acad. Sci. U.S.A.* **2019**, *116*, 23264–23273, doi:10.1073/pnas.1914557116.
21. Muller, F.L.; Colla, S.; Aquilanti, E.; Manzo, V.E.; Genovese, G.; Lee, J.; Eisenson, D.; Narurkar, R.; Deng, P.; Nezi, L.; et al. Passenger Deletions Generate Therapeutic Vulnerabilities in Cancer. *Nature* **2012**, *488*, 337–342, doi:10.1038/nature11331.
22. Lin, Y.-H.; Satani, N.; Hammoudi, N.; Yan, V.C.; Barekatain, Y.; Khadka, S.; Ackroyd, J.J.; Georgiou, D.K.; Pham, C.-D.; Arthur, K.; et al. An Enolase Inhibitor for the Targeted Treatment of ENO1-Deleted Cancers. *Nat Metab* **2020**, *2*, 1413–1426, doi:10.1038/s42255-020-00313-3.
23. Leonard, P.G.; Satani, N.; Maxwell, D.; Lin, Y.-H.; Hammoudi, N.; Peng, Z.; Pisaneschi, F.; Link, T.M.; Lee, G.R.; Sun, D.; et al. SF2312 Is a Natural Phosphonate Inhibitor of Enolase. *Nat Chem Biol* **2016**, *12*, 1053–1058, doi:10.1038/nchembio.2195.
24. Shang, D.; Wu, J.; Guo, L.; Xu, Y.; Liu, L.; Lu, J. Metformin Increases Sensitivity of Osteosarcoma Stem Cells to Cisplatin by Inhibiting Expression of PKM2. *International Journal of Oncology* **2017**, *50*, 1848–1856, doi:10.3892/ijo.2017.3950.
25. Su, Q.; Tao, T.; Tang, L.; Deng, J.; Darko, K.O.; Zhou, S.; Peng, M.; He, S.; Zeng, Q.; Chen, A.F.; et al. Down-regulation of PKM2 Enhances Anticancer Efficiency of THP on Bladder Cancer. *J Cell Mol Med* **2018**, *22*, 2774–2790, doi:10.1111/jcmm.13571.
26. Zhou, Y.; Huang, Z.; Su, J.; Li, J.; Zhao, S.; Wu, L.; Zhang, J.; He, Y.; Zhang, G.; Tao, J.; et al. Benserazide Is a Novel Inhibitor Targeting PKM2 for Melanoma Treatment. *Int. J. Cancer* **2020**, *147*, 139–151, doi:10.1002/ijc.32756.
27. Wang, X.; Zhang, F.; Wu, X.-R. Inhibition of Pyruvate Kinase M2 Markedly Reduces Chemoresistance of Advanced Bladder Cancer to Cisplatin. *Sci Rep* **2017**, *7*, 45983, doi:10.1038/srep45983.
28. Thonsri, U.; Seubwai, W.; Waraasawapati, S.; Wongkham, S.; Boonmars, T.; Cha'on, U.; Wongkham, C. Antitumor Effect of Shikonin, a PKM2 Inhibitor, in Cholangiocarcinoma Cell Lines. *Anticancer Res* **2020**, *40*, 5115–5124, doi:10.21873/anticanres.14515.
29. Tang, J.-C.; Zhao, J.; Long, F.; Chen, J.-Y.; Mu, B.; Jiang, Z.; Ren, Y.; Yang, J. Efficacy of Shikonin against Esophageal Cancer Cells and Its Possible Mechanisms in Vitro and in Vivo. *J Cancer* **2018**, *9*, 32–40, doi:10.7150/jca.21224.
30. Wu, H.; Wang, Y.; Wu, C.; Yang, P.; Li, H.; Li, Z. Resveratrol Induces Cancer Cell Apoptosis through MiR-326/PKM2-Mediated ER Stress and Mitochondrial Fission. *J. Agric. Food Chem.* **2016**, *64*, 9356–9367, doi:10.1021/acs.jafc.6b04549.
31. Zhao, H.; Han, L.; Jian, Y.; Ma, Y.; Yan, W.; Chen, X.; Xu, H.; Li, L. Resveratrol Induces Apoptosis in Human Melanoma Cell through Negatively Regulating Erk/PKM2/Bcl-2 Axis. *OTT* **2018**, *Volume 11*, 8995–9006, doi:10.2147/OTT.S186247.
32. Chen, J.; Jiang, Z.; Wang, B.; Wang, Y.; Hu, X. Vitamin K(3) and K(5) Are Inhibitors of Tumor Pyruvate Kinase M2. *Cancer Lett* **2012**, *316*, 204–210, doi:10.1016/j.canlet.2011.10.039.
33. Tang, W.; Liu, Z.; Mai, X.; Qi, X.; Li, D.; Gu, Q.; Li, J. Identification of Gliotoxin Isolated from Marine Fungus as a New Pyruvate Kinase M2 Inhibitor. *Biochemical and Biophysical Research Communications* **2020**, *528*, 594–600, doi:10.1016/j.bbrc.2020.05.139.

34. Ning, X.; Qi, H.; Li, R.; Li, Y.; Jin, Y.; McNutt, M.A.; Liu, J.; Yin, Y. Discovery of Novel Naphthoquinone Derivatives as Inhibitors of the Tumor Cell Specific M2 Isoform of Pyruvate Kinase. *European Journal of Medicinal Chemistry* **2017**, *138*, 343–352, doi:10.1016/j.ejmech.2017.06.064.
35. Zhao, Z.; Han, F.; Yang, S.; Wu, J.; Zhan, W. Oxamate-Mediated Inhibition of Lactate Dehydrogenase Induces Protective Autophagy in Gastric Cancer Cells: Involvement of the Akt-MTOR Signaling Pathway. *Cancer Lett* **2015**, *358*, 17–26, doi:10.1016/j.canlet.2014.11.046.
36. Valvona, C.J.; Fillmore, H.L. Oxamate, but Not Selective Targeting of LDH-A, Inhibits Medulloblastoma Cell Glycolysis, Growth and Motility. *Brain Sci* **2018**, *8*, E56, doi:10.3390/brainsci8040056.
37. Stone, S.C.; Rossetti, R.A.M.; Alvarez, K.L.F.; Carvalho, J.P.; Margarido, P.F.R.; Baracat, E.C.; Tacla, M.; Boccardo, E.; Yokochi, K.; Lorenzi, N.P.; et al. Lactate Secreted by Cervical Cancer Cells Modulates Macrophage Phenotype. *J Leukoc Biol* **2019**, *105*, 1041–1054, doi:10.1002/JLB.3A0718-274RR.
38. Cassim, S.; Raymond, V.-A.; Dehbidi-Assadzadeh, L.; Lapierre, P.; Bilodeau, M. Metabolic Reprogramming Enables Hepatocarcinoma Cells to Efficiently Adapt and Survive to a Nutrient-Restricted Microenvironment. *Cell Cycle* **2018**, *17*, 903–916, doi:10.1080/15384101.2018.1460023.
39. Qiao, T.; Xiong, Y.; Feng, Y.; Guo, W.; Zhou, Y.; Zhao, J.; Jiang, T.; Shi, C.; Han, Y. Inhibition of LDH-A by Oxamate Enhances the Efficacy of Anti-PD-1 Treatment in an NSCLC Humanized Mouse Model. *Front. Oncol.* **2021**, *11*, 632364, doi:10.3389/fonc.2021.632364.
40. Shelley, M.D.; Hartley, L.; Fish, R.G.; Groundwater, P.; Morgan, J.J.; Mort, D.; Mason, M.; Evans, A. Stereo-Specific Cytotoxic Effects of Gossypol Enantiomers and Gossypolone in Tumour Cell Lines. *Cancer Lett* **1999**, *135*, 171–180, doi:10.1016/s0304-3835(98)00302-4.
41. Coyle, T.; Levante, S.; Shetler, M.; Winfield, J. In Vitro and in Vivo Cytotoxicity of Gossypol against Central Nervous System Tumor Cell Lines. *J Neurooncol* **1994**, *19*, 25–35, doi:10.1007/BF01051046.
42. Gilbert, N.E.; O'Reilly, J.E.; Chang, C.J.G.; Lin, Y.C.; Brueggemeier, R.W. Antiproliferative Activity of Gossypol and Gossypolone on Human Breast Cancer Cells. *Life Sciences* **1995**, *57*, 61–67, doi:10.1016/0024-3205(95)00243-Y.
43. Volate, S.R.; Kawasaki, B.T.; Hurt, E.M.; Milner, J.A.; Kim, Y.S.; White, J.; Farrar, W.L. Gossypol Induces Apoptosis by Activating P53 in Prostate Cancer Cells and Prostate Tumor–Initiating Cells. *Mol Cancer Ther* **2010**, *9*, 461–470, doi:10.1158/1535-7163.MCT-09-0507.
44. Lan, L.; Appelman, C.; Smith, A.R.; Yu, J.; Larsen, S.; Marquez, R.T.; Liu, H.; Wu, X.; Gao, P.; Roy, A.; et al. Natural Product (−)-Gossypol Inhibits Colon Cancer Cell Growth by Targeting RNA-Binding Protein Musashi-1. *Molecular Oncology* **2015**, *9*, 1406–1420, doi:10.1016/j.molonc.2015.03.014.
45. Wolter, K.G.; Wang, S.J.; Henson, B.S.; Wang, S.; Griffith, K.A.; Kumar, B.; Chen, J.; Carey, T.E.; Bradford, C.R.; D'Silva, N.J. (−)-Gossypol Inhibits Growth and Promotes Apoptosis of Human Head and Neck Squamous Cell Carcinoma In Vivo. *Neoplasia* **2006**, *8*, 163–172, doi:10.1593/neo.05691.
46. Xian, Z.-Y.; Liu, J.-M.; Chen, Q.-K.; Chen, H.-Z.; Ye, C.-J.; Xue, J.; Yang, H.-Q.; Li, J.-L.; Liu, X.-F.; Kuang, S.-J. Inhibition of LDHA Suppresses Tumor Progression in Prostate Cancer. *Tumor Biol.* **2015**, *36*, 8093–8100, doi:10.1007/s13277-015-3540-x.
47. He, Y.; Chen, X.; Yu, Y.; Li, J.; Hu, Q.; Xue, C.; Chen, J.; Shen, S.; Luo, Y.; Ren, F.; et al. LDHA Is a Direct Target of MiR-30d-5p and Contributes to Aggressive Progression of Gallbladder Carcinoma. *Mol Carcinog* **2018**, *57*, 772–783, doi:10.1002/mc.22799.
48. Rellinger, E.J.; Craig, B.T.; Alvarez, A.L.; Dusek, H.L.; Kim, K.W.; Qiao, J.; Chung, D.H. FX11 Inhibits Aerobic Glycolysis and Growth of Neuroblastoma Cells. *Surgery* **2017**, *161*, 747–752, doi:10.1016/j.surg.2016.09.009.
49. Le, A.; Cooper, C.R.; Gouw, A.M.; Dinavahi, R.; Maitra, A.; Deck, L.M.; Royer, R.E.; Vander Jagt, D.L.; Semenza, G.L.; Dang, C.V. Inhibition of Lactate Dehydrogenase A Induces Oxidative Stress and Inhibits Tumor Progression. *Proc. Natl. Acad. Sci. U.S.A.* **2010**, *107*, 2037–2042, doi:10.1073/pnas.0914433107.
50. Billiard, J.; Dennison, J.B.; Briand, J.; Annan, R.S.; Chai, D.; Colón, M.; Dodson, C.S.; Gilbert, S.A.; Greshock, J.; Jing, J.; et al. Quinoline 3-Sulfonamides Inhibit Lactate Dehydrogenase A and Reverse Aerobic Glycolysis in Cancer Cells. *Cancer Metab* **2013**, *1*, 19, doi:10.1186/2049-3002-1-19.
51. Granchi, C.; Roy, S.; De Simone, A.; Salvetti, I.; Tuccinardi, T.; Martinelli, A.; Macchia, M.; Lanza, M.; Betti, L.; Giannaccini, G.; et al. N-Hydroxyindole-Based Inhibitors of Lactate Dehydrogenase

against Cancer Cell Proliferation. *European Journal of Medicinal Chemistry* **2011**, *46*, 5398–5407, doi:10.1016/j.ejmech.2011.08.046.
52. Granchi, C.; Roy, S.; Giacomelli, C.; Macchia, M.; Tuccinardi, T.; Martinelli, A.; Lanza, M.; Betti, L.; Giannaccini, G.; Lucacchini, A.; et al. Discovery of *N* -Hydroxyindole-Based Inhibitors of Human Lactate Dehydrogenase Isoform A (LDH-A) as Starvation Agents against Cancer Cells. *J. Med. Chem.* **2011**, *54*, 1599–1612, doi:10.1021/jm101007q.
53. Han, X.; Sheng, X.; Jones, H.M.; Jackson, A.L.; Kilgore, J.; Stine, J.E.; Schointuch, M.N.; Zhou, C.; Bae-Jump, V.L. Evaluation of the Anti-Tumor Effects of Lactate Dehydrogenase Inhibitor Galloflavin in Endometrial Cancer Cells. *J Hematol Oncol* **2015**, *8*, 2, doi:10.1186/s13045-014-0097-x.
54. Vettraino, M.; Manerba, M.; Govoni, M.; Di Stefano, G. Galloflavin Suppresses Lactate Dehydrogenase Activity and Causes MYC Downregulation in Burkitt Lymphoma Cells through NAD/NADH-Dependent Inhibition of Sirtuin-1. *Anti-Cancer Drugs* **2013**, *24*, 862–870, doi:10.1097/CAD.0b013e328363ae50.
55. Farabegoli, F.; Vettraino, M.; Manerba, M.; Fiume, L.; Roberti, M.; Di Stefano, G. Galloflavin, a New Lactate Dehydrogenase Inhibitor, Induces the Death of Human Breast Cancer Cells with Different Glycolytic Attitude by Affecting Distinct Signaling Pathways. *European Journal of Pharmaceutical Sciences* **2012**, *47*, 729–738, doi:10.1016/j.ejps.2012.08.012.
56. Manerba, M.; Di Ianni, L.; Govoni, M.; Roberti, M.; Recanatini, M.; Di Stefano, G. LDH Inhibition Impacts on Heat Shock Response and Induces Senescence of Hepatocellular Carcinoma Cells. *European Journal of Pharmaceutical Sciences* **2017**, *105*, 91–98, doi:10.1016/j.ejps.2017.05.015.
57. Curtis, N.J.; Mooney, L.; Hopcroft, L.; Michopoulos, F.; Whalley, N.; Zhong, H.; Murray, C.; Logie, A.; Revill, M.; Byth, K.F.; et al. Pre-Clinical Pharmacology of AZD3965, a Selective Inhibitor of MCT1: DLBCL, NHL and Burkitt's Lymphoma Anti-Tumor Activity. *Oncotarget* **2017**, *8*, 69219–69236, doi:10.18632/oncotarget.18215.
58. Quanz, M.; Bender, E.; Kopitz, C.; Grünewald, S.; Schlicker, A.; Schwede, W.; Eheim, A.; Toschi, L.; Neuhaus, R.; Richter, C.; et al. Preclinical Efficacy of the Novel Monocarboxylate Transporter 1 Inhibitor BAY-8002 and Associated Markers of Resistance. *Mol Cancer Ther* **2018**, *17*, 2285–2296, doi:10.1158/1535-7163.MCT-17-1253.
59. Korga, A.; Ostrowska, M.; Iwan, M.; Herbet, M.; Dudka, J. Inhibition of Glycolysis Disrupts Cellular Antioxidant Defense and Sensitizes HepG2 Cells to Doxorubicin Treatment. *FEBS Open Bio* **2019**, *9*, 959–972, doi:10.1002/2211-5463.12628.
60. Sun, H.; Zhu, A.; Zhou, X.; Wang, F. Suppression of Pyruvate Dehydrogenase Kinase-2 Re-Sensitizes Paclitaxel-Resistant Human Lung Cancer Cells to Paclitaxel. *Oncotarget* **2017**, *8*, 52642–52650, doi:10.18632/oncotarget.16991.
61. Woolbright, B.L.; Choudhary, D.; Mikhalyuk, A.; Trammel, C.; Shanmugam, S.; Abbott, E.; Pilbeam, C.C.; Taylor, J.A. The Role of Pyruvate Dehydrogenase Kinase-4 (PDK4) in Bladder Cancer and Chemoresistance. *Mol Cancer Ther* **2018**, *17*, 2004–2012, doi:10.1158/1535-7163.MCT-18-0063.
62. Fang, Z.; Jiang, C.; Feng, Y.; Chen, R.; Lin, X.; Zhang, Z.; Han, L.; Chen, X.; Li, H.; Guo, Y.; et al. Effects of G6PD Activity Inhibition on the Viability, ROS Generation and Mechanical Properties of Cervical Cancer Cells. *Biochimica et Biophysica Acta (BBA) - Molecular Cell Research* **2016**, *1863*, 2245–2254, doi:10.1016/j.bbamcr.2016.05.016.
63. Catanzaro, D.; Gaude, E.; Orso, G.; Giordano, C.; Guzzo, G.; Rasola, A.; Ragazzi, E.; Caparrotta, L.; Frezza, C.; Montopoli, M. Inhibition of Glucose-6-Phosphate Dehydrogenase Sensitizes Cisplatin-Resistant Cells to Death. *Oncotarget* **2015**, *6*, 30102–30114, doi:10.18632/oncotarget.4945.
64. Xu, Y.; Gao, W.; Zhang, Y.; Wu, S.; Liu, Y.; Deng, X.; Xie, L.; Yang, J.; Yu, H.; Su, J.; et al. ABT737 Reverses Cisplatin Resistance by Targeting Glucose Metabolism of Human Ovarian Cancer Cells. *Int J Oncol* **2018**, doi:10.3892/ijo.2018.4476.
65. Lucarelli, G.; Galleggiante, V.; Rutigliano, M.; Sanguedolce, F.; Cagiano, S.; Bufo, P.; Lastilla, G.; Maiorano, E.; Ribatti, D.; Giglio, A.; et al. Metabolomic Profile of Glycolysis and the Pentose Phosphate Pathway Identifies the Central Role of Glucose-6-Phosphate Dehydrogenase in Clear Cell-Renal Cell Carcinoma. *Oncotarget* **2015**, *6*, 13371–13386, doi:10.18632/oncotarget.3823.
66. Hong, W.; Cai, P.; Xu, C.; Cao, D.; Yu, W.; Zhao, Z.; Huang, M.; Jin, J. Inhibition of Glucose-6-Phosphate Dehydrogenase Reverses Cisplatin Resistance in Lung Cancer Cells via the Redox System. *Front. Pharmacol.* **2018**, *9*, 43, doi:10.3389/fphar.2018.00043.

67. Chen, X.; Xu, Z.; Zhu, Z.; Chen, A.; Fu, G.; Wang, Y.; Pan, H.; Jin, B. Modulation of G6PD Affects Bladder Cancer via ROS Accumulation and the AKT Pathway in Vitro. *Int J Oncol* **2018**, doi:10.3892/ijo.2018.4501.
68. Arbe, M.F.; Agnetti, L.; Breininger, E.; Glikin, G.C.; Finocchiaro, L.M.E.; Villaverde, M.S. Glucose 6-Phosphate Dehydrogenase Inhibition Sensitizes Melanoma Cells to Metformin Treatment. *Translational Oncology* **2020**, *13*, 100842, doi:10.1016/j.tranon.2020.100842.
69. Liu, H.; Zhao, S.; Zhang, Y.; Wu, J.; Peng, H.; Fan, J.; Liao, J. Reactive Oxygen Species-Mediated Endoplasmic Reticulum Stress and Mitochondrial Dysfunction Contribute to Polydatin-Induced Apoptosis in Human Nasopharyngeal Carcinoma CNE Cells. *J. Cell. Biochem.* **2011**, *112*, 3695–3703, doi:10.1002/jcb.23303.
70. Wang, C.; Luo, Y.; Lu, J.; Wang, Y.; Sheng, G. Polydatin Induces Apoptosis and Inhibits Growth of Acute Monocytic Leukemia Cells: EFFECT OF PD ON THP-1 CELLS. *J Biochem Mol Toxicol* **2016**, *30*, 200–205, doi:10.1002/jbt.21779.
71. Mele, L.; Paino, F.; Papaccio, F.; Regad, T.; Boocock, D.; Stiuso, P.; Lombardi, A.; Liccardo, D.; Aquino, G.; Barbieri, A.; et al. A New Inhibitor of Glucose-6-Phosphate Dehydrogenase Blocks Pentose Phosphate Pathway and Suppresses Malignant Proliferation and Metastasis in Vivo. *Cell Death Dis* **2018**, *9*, 572, doi:10.1038/s41419-018-0635-5.
72. Jiao, Y.; Wu, Y.; Du, D. Polydatin Inhibits Cell Proliferation, Invasion and Migration, and Induces Cell Apoptosis in Hepatocellular Carcinoma. *Braz J Med Biol Res* **2018**, *51*, e6867, doi:10.1590/1414-431x20176867.
73. Wang, X.; Wu, G.; Cao, G.; Yang, L.; Xu, H.; Huang, J.; Hou, J. Zoledronic Acid Inhibits the Pentose Phosphate Pathway through Attenuating the Ras-TAp73-G6PD Axis in Bladder Cancer Cells. *Molecular Medicine Reports* **2015**, *12*, 4620–4625, doi:10.3892/mmr.2015.3995.
74. Hanaford, A.R.; Alt, J.; Rais, R.; Wang, S.Z.; Kaur, H.; Thorek, D.L.J.; Eberhart, C.G.; Slusher, B.S.; Martin, A.M.; Raabe, E.H. Orally Bioavailable Glutamine Antagonist Prodrug JHU-083 Penetrates Mouse Brain and Suppresses the Growth of MYC-Driven Medulloblastoma. *Transl Oncol* **2019**, *12*, 1314–1322, doi:10.1016/j.tranon.2019.05.013.
75. Seltzer, M.J.; Bennett, B.D.; Joshi, A.D.; Gao, P.; Thomas, A.G.; Ferraris, D.V.; Tsukamoto, T.; Rojas, C.J.; Slusher, B.S.; Rabinowitz, J.D.; et al. Inhibition of Glutaminase Preferentially Slows Growth of Glioma Cells with Mutant IDH1. *Cancer Res* **2010**, *70*, 8981–8987, doi:10.1158/0008-5472.CAN-10-1666.
76. Le, A.; Lane, A.N.; Hamaker, M.; Bose, S.; Gouw, A.; Barbi, J.; Tsukamoto, T.; Rojas, C.J.; Slusher, B.S.; Zhang, H.; et al. Glucose-Independent Glutamine Metabolism via TCA Cycling for Proliferation and Survival in B-Cells. *Cell Metab* **2012**, *15*, 110–121, doi:10.1016/j.cmet.2011.12.009.
77. Son, J.; Lyssiotis, C.A.; Ying, H.; Wang, X.; Hua, S.; Ligorio, M.; Perera, R.M.; Ferrone, C.R.; Mullarky, E.; Shyh-Chang, N.; et al. Glutamine Supports Pancreatic Cancer Growth through a KRAS-Regulated Metabolic Pathway. *Nature* **2013**, *496*, 101–105, doi:10.1038/nature12040.
78. Qie, S.; Chu, C.; Li, W.; Wang, C.; Sang, N. ErbB2 Activation Upregulates Glutaminase 1 Expression Which Promotes Breast Cancer Cell Proliferation. *J Cell Biochem* **2014**, *115*, 498–509, doi:10.1002/jcb.24684.
79. van den Heuvel, A.P.J.; Jing, J.; Wooster, R.F.; Bachman, K.E. Analysis of Glutamine Dependency in Non-Small Cell Lung Cancer: GLS1 Splice Variant GAC Is Essential for Cancer Cell Growth. *Cancer Biology & Therapy* **2012**, *13*, 1185–1194, doi:10.4161/cbt.21348.
80. Gross, M.I.; Demo, S.D.; Dennison, J.B.; Chen, L.; Chernov-Rogan, T.; Goyal, B.; Janes, J.R.; Laidig, G.J.; Lewis, E.R.; Li, J.; et al. Antitumor Activity of the Glutaminase Inhibitor CB-839 in Triple-Negative Breast Cancer. *Mol Cancer Ther* **2014**, *13*, 890–901, doi:10.1158/1535-7163.MCT-13-0870.
81. Ruan, J.J.; Yu, Y.; Hou, W.; Chen, Z.; Fang, J.; Zhang, J.; Ni, M.; Li, D.; Lu, S.; Rui, J.; et al. Kidney-Type Glutaminase Inhibitor Hexylselen Selectively Kills Cancer Cells via a Three-Pronged Mechanism. *ACS Pharmacol. Transl. Sci.* **2019**, *2*, 18–30, doi:10.1021/acsptsci.8b00047.
82. Matre, P.; Shariati, M.; Velez, J.; Qi, Y.; Konoplev, S.; Su, X.; DiNardo, C.D.; Daver, N.; Majeti, R.; Andreeff, M.; et al. Efficacy of Novel Glutaminase Inhibitor CB-839 in Acute Myeloid Leukemia. *Blood* **2014**, *124*, 3763–3763, doi:10.1182/blood.V124.21.3763.3763.

83. Xie, C.; Jin, J.; Bao, X.; Zhan, W.-H.; Han, T.-Y.; Gan, M.; Zhang, C.; Wang, J. Inhibition of Mitochondrial Glutaminase Activity Reverses Acquired Erlotinib Resistance in Non-Small Cell Lung Cancer. *Oncotarget* **2015**, *7*, 610–621.
84. Kahlert, U.D.; Cheng, M.; Koch, K.; Marchionni, L.; Fan, X.; Raabe, E.H.; Maciaczyk, J.; Glunde, K.; Eberhart, C.G. Alterations in Cellular Metabolome after Pharmacological Inhibition of Notch in Glioblastoma Cells. *Int J Cancer* **2016**, *138*, 1246–1255, doi:10.1002/ijc.29873.
85. Yuan, L.; Sheng, X.; Clark, L.H.; Zhang, L.; Guo, H.; Jones, H.M.; Willson, A.K.; Gehrig, P.A.; Zhou, C.; Bae-Jump, V.L. Glutaminase Inhibitor Compound 968 Inhibits Cell Proliferation and Sensitizes Paclitaxel in Ovarian Cancer. *Am J Transl Res* **2016**, *8*, 4265–4277.
86. Zhang, J.; Wang, G.; Mao, Q.; Li, S.; Xiong, W.; Lin, Y.; Ge, J. Glutamate Dehydrogenase (GDH) Regulates Bioenergetics and Redox Homeostasis in Human Glioma. *Oncotarget* **2016**, *5*, doi:10.18632/oncotarget.7657.
87. Jin, L.; Li, D.; Alesi, G.N.; Fan, J.; Kang, H.-B.; Lu, Z.; Boggon, T.J.; Jin, P.; Yi, H.; Wright, E.R.; et al. Glutamate Dehydrogenase 1 Signals through Antioxidant Glutathione Peroxidase 1 to Regulate Redox Homeostasis and Tumor Growth. *Cancer Cell* **2015**, *27*, 257–270, doi:10.1016/j.ccell.2014.12.006.
88. Hassanein, M.; Hoeksema, M.D.; Shiota, M.; Qian, J.; Harris, B.K.; Chen, H.; Clark, J.E.; Alborn, W.E.; Eisenberg, R.; Massion, P.P. SLC1A5 Mediates Glutamine Transport Required for Lung Cancer Cell Growth and Survival. *Clin Cancer Res* **2013**, *19*, 560–570, doi:10.1158/1078-0432.CCR-12-2334.
89. Ren, P.; Yue, M.; Xiao, D.; Xiu, R.; Gan, L.; Liu, H.; Qing, G. ATF4 and N-Myc Coordinate Glutamine Metabolism in *MYCN* -Amplified Neuroblastoma Cells through ASCT2 Activation. *J. Pathol.* **2015**, *235*, 90–100, doi:10.1002/path.4429.
90. Wang, Q.; Hardie, R.; Hoy, A.J.; van Geldermalsen, M.; Gao, D.; Fazli, L.; Sadowski, M.C.; Balaban, S.; Schreuder, M.; Nagarajah, R.; et al. Targeting ASCT2 -mediated Glutamine Uptake Blocks Prostate Cancer Growth and Tumour Development. *J. Pathol.* **2015**, *236*, 278–289, doi:10.1002/path.4518.
91. van Geldermalsen, M.; Wang, Q.; Nagarajah, R.; Marshall, A.D.; Thoeng, A.; Gao, D.; Ritchie, W.; Feng, Y.; Bailey, C.G.; Deng, N.; et al. ASCT2/SLC1A5 Controls Glutamine Uptake and Tumour Growth in Triple-Negative Basal-like Breast Cancer. *Oncogene* **2016**, *35*, 3201–3208, doi:10.1038/onc.2015.381.
92. Marshall, A.D.; van Geldermalsen, M.; Otte, N.J.; Lum, T.; Vellozzi, M.; Thoeng, A.; Pang, A.; Nagarajah, R.; Zhang, B.; Wang, Q.; et al. ASCT2 Regulates Glutamine Uptake and Cell Growth in Endometrial Carcinoma. *Oncogenesis* **2017**, *6*, e367, doi:10.1038/oncsis.2017.70.
93. Schulte, M.L.; Fu, A.; Zhao, P.; Li, J.; Geng, L.; Smith, S.T.; Kondo, J.; Coffey, R.J.; Johnson, M.O.; Rathmell, J.C.; et al. Pharmacological Blockade of ASCT2-Dependent Glutamine Transport Leads to Antitumor Efficacy in Preclinical Models. *Nat Med* **2018**, *24*, 194–202, doi:10.1038/nm.4464.
94. van Geldermalsen, M.; Quek, L.-E.; Turner, N.; Freidman, N.; Pang, A.; Guan, Y.F.; Krycer, J.R.; Ryan, R.; Wang, Q.; Holst, J. Benzylserine Inhibits Breast Cancer Cell Growth by Disrupting Intracellular Amino Acid Homeostasis and Triggering Amino Acid Response Pathways. *BMC Cancer* **2018**, *18*, 689, doi:10.1186/s12885-018-4599-8.
95. Sun, H.-J.; Meng, L.-Y.; Shen, Y.; Zhu, Y.-Z.; Liu, H.-R. S-Benzyl-Cysteine-Mediated Cell Cycle Arrest and Apoptosis Involving Activation of Mitochondrial-Dependent Caspase Cascade through the P53 Pathway in Human Gastric Cancer SGC-7901 Cells. *Asian Pacific Journal of Cancer Prevention* **2013**, *14*, 6379–6384, doi:10.7314/APJCP.2013.14.11.6379.
96. Schlaepfer, I.R.; Rider, L.; Rodrigues, L.U.; Gijón, M.A.; Pac, C.T.; Romero, L.; Cimic, A.; Sirintrapun, S.J.; Glodé, L.M.; Eckel, R.H.; et al. Lipid Catabolism via CPT1 as a Therapeutic Target for Prostate Cancer. *Mol Cancer Ther* **2014**, *13*, 2361–2371, doi:10.1158/1535-7163.MCT-14-0183.
97. Tan, Z.; Xiao, L.; Tang, M.; Bai, F.; Li, J.; Li, L.; Shi, F.; Li, N.; Li, Y.; Du, Q.; et al. Targeting CPT1A-Mediated Fatty Acid Oxidation Sensitizes Nasopharyngeal Carcinoma to Radiation Therapy. *Theranostics* **2018**, *8*, 2329–2347, doi:10.7150/thno.21451.
98. Samudio, I.; Harmancey, R.; Fiegl, M.; Kantarjian, H.; Konopleva, M.; Korchin, B.; Kaluarachchi, K.; Bornmann, W.; Duvvuri, S.; Taegtmeyer, H.; et al. Pharmacologic Inhibition of Fatty Acid Oxidation

Sensitizes Human Leukemia Cells to Apoptosis Induction. *J Clin Invest* **2010**, *120*, 142–156, doi:10.1172/JCI38942.
99. Jariwala, N.; Mehta, G.A.; Bhatt, V.; Hussein, S.; Parker, K.A.; Yunus, N.; Parker, J.S.; Guo, J.Y.; Gatza, M.L. CPT1A and Fatty Acid β-Oxidation Are Essential for Tumor Cell Growth and Survival in Hormone Receptor-Positive Breast Cancer. *NAR Cancer* **2021**, *3*, zcab035, doi:10.1093/narcan/zcab035.
100. Pacilli, A.; Calienni, M.; Margarucci, S.; D'Apolito, M.; Petillo, O.; Rocchi, L.; Pasquinelli, G.; Nicolai, R.; Koverech, A.; Calvani, M.; et al. Carnitine-Acyltransferase System Inhibition, Cancer Cell Death, and Prevention of Myc-Induced Lymphomagenesis. *JNCI: Journal of the National Cancer Institute* **2013**, *105*, 489–498, doi:10.1093/jnci/djt030.
101. Ricciardi, M.R.; Mirabilii, S.; Allegretti, M.; Licchetta, R.; Calarco, A.; Torrisi, M.R.; Foà, R.; Nicolai, R.; Peluso, G.; Tafuri, A. Targeting the Leukemia Cell Metabolism by the CPT1a Inhibition: Functional Preclinical Effects in Leukemias. *Blood* **2015**, *126*, 1925–1929, doi:10.1182/blood-2014-12-617498.
102. Mascagna, D.; Ghanem, G.; Morandini, R.; d'Ischia, M.; Misuraca, G.; Lejeune, F.; Prota, G. Synthesis and Cytotoxic Properties of New N-Substituted 4-Aminophenol Derivatives with a Potential as Antimelanoma Agents. *Melanoma Res* **1992**, *2*, 25–32, doi:10.1097/00008390-199205000-00004.
103. Lee, E.A.; Angka, L.; Rota, S.-G.; Hanlon, T.; Mitchell, A.; Hurren, R.; Wang, X.M.; Gronda, M.; Boyaci, E.; Bojko, B.; et al. Targeting Mitochondria with Avocatin B Induces Selective Leukemia Cell Death. *Cancer Research* **2015**, *75*, 2478–2488, doi:10.1158/0008-5472.CAN-14-2676.
104. Liu, P.; Liu, J.; Jiang, W.; Carew, J.S.; Ogasawara, M.A.; Pelicano, H.; Croce, C.M.; Estrov, Z.; Xu, R.; Keating, M.J.; et al. Elimination of Chronic Lymphocytic Leukemia Cells in Stromal Microenvironment by Targeting CPT with an Anti-Angina Drug Perhexiline. *Oncogene* **2016**, *35*, 5663–5673, doi:10.1038/onc.2016.103.
105. Mims, J.; Bansal, N.; Bharadwaj, M.S.; Chen, X.; Molina, A.J.; Tsang, A.W.; Furdui, C.M. Energy Metabolism in a Matched Model of Radiation Resistance for Head and Neck Squamous Cell Cancer. *Radiation Research* **2015**, *183*, 291–304, doi:10.1667/RR13828.1.
106. Grube, S.; Dünisch, P.; Freitag, D.; Klausnitzer, M.; Sakr, Y.; Walter, J.; Kalff, R.; Ewald, C. Overexpression of Fatty Acid Synthase in Human Gliomas Correlates with the WHO Tumor Grade and Inhibition with Orlistat Reduces Cell Viability and Triggers Apoptosis. *J Neurooncol* **2014**, *118*, 277–287, doi:10.1007/s11060-014-1452-z.
107. Czumaj, A.; Zabielska, J.; Pakiet, A.; Mika, A.; Rostkowska, O.; Makarewicz, W.; Kobiela, J.; Sledzinski, T.; Stelmanska, E. *In Vivo* Effectiveness of Orlistat in the Suppression of Human Colorectal Cancer Cell Proliferation. *Anticancer Res* **2019**, *39*, 3815–3822, doi:10.21873/anticanres.13531.
108. Chuang, H.-Y.; Lee, Y.-P.; Lin, W.-C.; Lin, Y.-H.; Hwang, J.-J. Fatty Acid Inhibition Sensitizes Androgen-Dependent and -Independent Prostate Cancer to Radiotherapy via FASN/NF-KB Pathway. *Sci Rep* **2019**, *9*, 13284, doi:10.1038/s41598-019-49486-2.
109. Sokolowska, E.; Presler, M.; Goyke, E.; Milczarek, R.; Swierczynski, J.; Sledzinski, T. Orlistat Reduces Proliferation and Enhances Apoptosis in Human Pancreatic Cancer Cells (PANC-1). *Anticancer Res* **2017**, *37*, 6321–6327, doi:10.21873/anticanres.12083.
110. You, B.-J.; Chen, L.-Y.; Hsu, P.-H.; Sung, P.-H.; Hung, Y.-C.; Lee, H.-Z. Orlistat Displays Antitumor Activity and Enhances the Efficacy of Paclitaxel in Human Hepatoma Hep3B Cells. *Chem Res Toxicol* **2019**, *32*, 255–264, doi:10.1021/acs.chemrestox.8b00269.
111. Li, C.-F.; Fang, F.-M.; Chen, Y.-Y.; Liu, T.-T.; Chan, T.-C.; Yu, S.-C.; Chen, L.-T.; Huang, H.-Y. Overexpressed Fatty Acid Synthase in Gastrointestinal Stromal Tumors: Targeting a Progression-Associated Metabolic Driver Enhances the Antitumor Effect of Imatinib. *Clin Cancer Res* **2017**, *23*, 4908–4918, doi:10.1158/1078-0432.CCR-16-2770.
112. Kant, S.; Kumar, A.; Singh, S.M. Fatty Acid Synthase Inhibitor Orlistat Induces Apoptosis in T Cell Lymphoma: Role of Cell Survival Regulatory Molecules. *Biochimica et Biophysica Acta (BBA) - General Subjects* **2012**, *1820*, 1764–1773, doi:10.1016/j.bbagen.2012.07.010.
113. Carvalho, M.A.; Zecchin, K.G.; Seguin, F.; Bastos, D.C.; Agostini, M.; Rangel, A.L.C.A.; Veiga, S.S.; Raposo, H.F.; Oliveira, H.C.F.; Loda, M.; et al. Fatty Acid Synthase Inhibition with Orlistat Promotes

Apoptosis and Reduces Cell Growth and Lymph Node Metastasis in a Mouse Melanoma Model. *Int. J. Cancer* **2008**, *123*, 2557–2565, doi:10.1002/ijc.23835.
114. Papaevangelou, E.; Almeida, G.S.; Box, C.; deSouza, N.M.; Chung, Y.-L. The Effect of FASN Inhibition on the Growth and Metabolism of a Cisplatin-Resistant Ovarian Carcinoma Model: FASN Inhibition in Cisplatin-Resistant Cancer. *Int. J. Cancer* **2018**, *143*, 992–1002, doi:10.1002/ijc.31392.
115. Pizer, E.S.; Jackisch, C.; Wood, F.D.; Pasternack, G.R.; Davidson, N.E.; Kuhajda, F.P. Inhibition of Fatty Acid Synthesis Induces Programmed Cell Death in Human Breast Cancer Cells. *Cancer Res* **1996**, *56*, 2745–2747.
116. Chang, L.; Wu, P.; Senthilkumar, R.; Tian, X.; Liu, H.; Shen, X.; Tao, Z.; Huang, P. Loss of Fatty Acid Synthase Suppresses the Malignant Phenotype of Colorectal Cancer Cells by Down-Regulating Energy Metabolism and MTOR Signaling Pathway. *J Cancer Res Clin Oncol* **2016**, *142*, 59–72, doi:10.1007/s00432-015-2000-8.
117. Deepa, P.R.; Vandhana, S.; Jayanthi, U.; Krishnakumar, S. Therapeutic and Toxicologic Evaluation of Anti-Lipogenic Agents in Cancer Cells Compared with Non-Neoplastic Cells. *Basic Clin Pharmacol Toxicol* **2012**, *110*, 494–503, doi:10.1111/j.1742-7843.2011.00844.x.
118. Gouw, A.M.; Eberlin, L.S.; Margulis, K.; Sullivan, D.K.; Toal, G.G.; Tong, L.; Zare, R.N.; Felsher, D.W. Oncogene KRAS Activates Fatty Acid Synthase, Resulting in Specific ERK and Lipid Signatures Associated with Lung Adenocarcinoma. *Proc Natl Acad Sci U S A* **2017**, *114*, 4300–4305, doi:10.1073/pnas.1617709114.
119. Jiang, B.; Li, E.-H.; Lu, Y.-Y.; Jiang, Q.; Cui, D.; Jing, Y.-F.; Xia, S.-J. Inhibition of Fatty-Acid Synthase Suppresses P-AKT and Induces Apoptosis in Bladder Cancer. *Urology* **2012**, *80*, 484.e9-15, doi:10.1016/j.urology.2012.02.046.
120. Pizer, E.S.; Wood, F.D.; Heine, H.S.; Romantsev, F.E.; Pasternack, G.R.; Kuhajda, F.P. Inhibition of Fatty Acid Synthesis Delays Disease Progression in a Xenograft Model of Ovarian Cancer. *Cancer Res* **1996**, *56*, 1189–1193.
121. Shiragami, R.; Murata, S.; Kosugi, C.; Tezuka, T.; Yamazaki, M.; Hirano, A.; Yoshimura, Y.; Suzuki, M.; Shuto, K.; Koda, K. Enhanced Antitumor Activity of Cerulenin Combined with Oxaliplatin in Human Colon Cancer Cells. *Int J Oncol* **2013**, *43*, 431–438, doi:10.3892/ijo.2013.1978.
122. Rae, C.; Fragkoulis, G.I.; Chalmers, A.J. Cytotoxicity and Radiosensitizing Activity of the Fatty Acid Synthase Inhibitor C75 Is Enhanced by Blocking Fatty Acid Uptake in Prostate Cancer Cells. *Advances in Radiation Oncology* **2020**, *5*, 994–1005, doi:10.1016/j.adro.2020.06.022.
123. Gao, Y.; Lin, L.-P.; Zhu, C.-H.; Chen, Y.; Hou, Y.-T.; Ding, J. Growth Arrest Induced by C75, A Fatty Acid Synthase Inhibitor, Was Partially Modulated by P38 MAPK but Not by P53 in Human Hepatocellular Carcinoma. *Cancer Biology & Therapy* **2006**, *5*, 978–985, doi:10.4161/cbt.5.8.2883.
124. Zaytseva, Y.Y.; Rychahou, P.G.; Le, A.-T.; Scott, T.L.; Flight, R.M.; Kim, J.T.; Harris, J.; Liu, J.; Wang, C.; Morris, A.J.; et al. Preclinical Evaluation of Novel Fatty Acid Synthase Inhibitors in Primary Colorectal Cancer Cells and a Patient-Derived Xenograft Model of Colorectal Cancer. *Oncotarget* **2018**, *9*, 24787–24800, doi:10.18632/oncotarget.25361.
125. Ventura, R.; Mordec, K.; Waszczuk, J.; Wang, Z.; Lai, J.; Fridlib, M.; Buckley, D.; Kemble, G.; Heuer, T.S. Inhibition of de Novo Palmitate Synthesis by Fatty Acid Synthase Induces Apoptosis in Tumor Cells by Remodeling Cell Membranes, Inhibiting Signaling Pathways, and Reprogramming Gene Expression. *EBioMedicine* **2015**, *2*, 808–824, doi:10.1016/j.ebiom.2015.06.020.
126. Tao, T.; Su, Q.; Xu, S.; Deng, J.; Zhou, S.; Zhuang, Y.; Huang, Y.; He, C.; He, S.; Peng, M.; et al. Down-regulation of PKM2 Decreases FASN Expression in Bladder Cancer Cells through AKT/MTOR/SREBP-1c Axis. *J Cell Physiol* **2019**, *234*, 3088–3104, doi:10.1002/jcp.27129.
127. Wheaton, W.W.; Weinberg, S.E.; Hamanaka, R.B.; Soberanes, S.; Sullivan, L.B.; Anso, E.; Glasauer, A.; Dufour, E.; Mutlu, G.M.; Budigner, G.S.; et al. Metformin Inhibits Mitochondrial Complex I of Cancer Cells to Reduce Tumorigenesis. *eLife* **2014**, *3*, e02242, doi:10.7554/eLife.02242.
128. Izreig, S.; Gariepy, A.; Kaymak, I.; Bridges, H.R.; Donayo, A.O.; Bridon, G.; DeCamp, L.M.; Kitchen-Goosen, S.M.; Avizonis, D.; Sheldon, R.D.; et al. Repression of LKB1 by MiR-17~92 Sensitizes MYC-Dependent Lymphoma to Biguanide Treatment. *Cell Rep Med* **2020**, *1*, 100014, doi:10.1016/j.xcrm.2020.100014.

129. Villani, L.A.; Smith, B.K.; Marcinko, K.; Ford, R.J.; Broadfield, L.A.; Green, A.E.; Houde, V.P.; Muti, P.; Tsakiridis, T.; Steinberg, G.R. The Diabetes Medication Canagliflozin Reduces Cancer Cell Proliferation by Inhibiting Mitochondrial Complex-I Supported Respiration. *Mol Metab* **2016**, *5*, 1048–1056, doi:10.1016/j.molmet.2016.08.014.
130. Schöckel, L.; Glasauer, A.; Basit, F.; Bitschar, K.; Truong, H.; Erdmann, G.; Algire, C.; Hägebarth, A.; Willems, P.H.; Kopitz, C.; et al. Targeting Mitochondrial Complex I Using BAY 87-2243 Reduces Melanoma Tumor Growth. *Cancer & Metabolism* **2015**, *3*, 11, doi:10.1186/s40170-015-0138-0.
131. Akatsuka, A.; Kojima, N.; Okamura, M.; Dan, S.; Yamori, T. A Novel Thiophene-3-Carboxamide Analog of Annonaceous Acetogenin Exhibits Antitumor Activity via Inhibition of Mitochondrial Complex I. *Pharmacol Res Perspect* **2016**, *4*, e00246, doi:10.1002/prp2.246.
132. Chen, G.; Zhang, X.; Zhao, M.; Wang, Y.; Cheng, X.; Wang, D.; Xu, Y.; Du, Z.; Yu, X. Celastrol Targets Mitochondrial Respiratory Chain Complex I to Induce Reactive Oxygen Species-Dependent Cytotoxicity in Tumor Cells. *BMC Cancer* **2011**, *11*, 170, doi:10.1186/1471-2407-11-170.
133. Bastian, A.; Matsuzaki, S.; Humphries, K.M.; Pharaoh, G.A.; Doshi, A.; Zaware, N.; Gangjee, A.; Ihnat, M.A. AG311, a Small Molecule Inhibitor of Complex I and Hypoxia-Induced HIF-1α Stabilization. *Cancer Lett* **2017**, *388*, 149–157, doi:10.1016/j.canlet.2016.11.040.
134. Zhang, B.; Chu, W.; Wei, P.; Liu, Y.; Wei, T. Xanthohumol Induces Generation of Reactive Oxygen Species and Triggers Apoptosis through Inhibition of Mitochondrial Electron Transfer Chain Complex I. *Free Radic Biol Med* **2015**, *89*, 486–497, doi:10.1016/j.freeradbiomed.2015.09.021.
135. Morgan, J.B.; Liu, Y.; Coothankandaswamy, V.; Mahdi, F.; Jekabsons, M.B.; Gerwick, W.H.; Valeriote, F.A.; Zhou, Y.-D.; Nagle, D.G. Kalkitoxin Inhibits Angiogenesis, Disrupts Cellular Hypoxic Signaling, and Blocks Mitochondrial Electron Transport in Tumor Cells. *Mar Drugs* **2015**, *13*, 1552–1568, doi:10.3390/md13031552.
136. Molina, J.R.; Sun, Y.; Protopopova, M.; Gera, S.; Bandi, M.; Bristow, C.; McAfoos, T.; Morlacchi, P.; Ackroyd, J.; Agip, A.-N.A.; et al. An Inhibitor of Oxidative Phosphorylation Exploits Cancer Vulnerability. *Nat Med* **2018**, *24*, 1036–1046, doi:10.1038/s41591-018-0052-4.
137. Stephenson, Z.A.; Harvey, R.F.; Pryde, K.R.; Mistry, S.; Hardy, R.E.; Serreli, R.; Chung, I.; Allen, T.E.; Stoneley, M.; MacFarlane, M.; et al. Identification of a Novel Toxicophore in Anti-Cancer Chemotherapeutics That Targets Mitochondrial Respiratory Complex I. *Elife* **2020**, *9*, e55845, doi:10.7554/eLife.55845.
138. Baccelli, I.; Gareau, Y.; Lehnertz, B.; Gingras, S.; Spinella, J.-F.; Corneau, S.; Mayotte, N.; Girard, S.; Frechette, M.; Blouin-Chagnon, V.; et al. Mubritinib Targets the Electron Transport Chain Complex I and Reveals the Landscape of OXPHOS Dependency in Acute Myeloid Leukemia. *Cancer Cell* **2019**, *36*, 84-99.e8, doi:10.1016/j.ccell.2019.06.003.
139. Cornelissen, J.; Wanders, R.J.; Van Gennip, A.H.; Van den Bogert, C.; Voûte, P.A.; Van Kuilenburg, A.B. Meta-Iodobenzylguanidine Inhibits Complex I and III of the Respiratory Chain in the Human Cell Line Molt-4. *Biochem Pharmacol* **1995**, *49*, 471–477, doi:10.1016/0006-2952(94)00450-z.
140. Thomas, A.G.; Rojas, C.; Tanega, C.; Shen, M.; Simeonov, A.; Boxer, M.B.; Auld, D.S.; Ferraris, D.V.; Tsukamoto, T.; Slusher, B.S. Kinetic Characterization of Ebselen, Chelerythrine and Apomorphine as Glutaminase Inhibitors. *Biochemical and Biophysical Research Communications* **2013**, *438*, 243–248, doi:10.1016/j.bbrc.2013.06.110.
141. Dong, L.-F.; Low, P.; Dyason, J.C.; Wang, X.-F.; Prochazka, L.; Witting, P.K.; Freeman, R.; Swettenham, E.; Valis, K.; Liu, J.; et al. Alpha-Tocopheryl Succinate Induces Apoptosis by Targeting Ubiquinone-Binding Sites in Mitochondrial Respiratory Complex II. *Oncogene* **2008**, *27*, 4324–4335, doi:10.1038/onc.2008.69.
142. Dong, L.-F.; Jameson, V.J.A.; Tilly, D.; Cerny, J.; Mahdavian, E.; Marín-Hernández, A.; Hernández-Esquivel, L.; Rodríguez-Enríquez, S.; Stursa, J.; Witting, P.K.; et al. Mitochondrial Targeting of Vitamin E Succinate Enhances Its Pro-Apoptotic and Anti-Cancer Activity via Mitochondrial Complex II. *J Biol Chem* **2011**, *286*, 3717–3728, doi:10.1074/jbc.M110.186643.
143. Wang, H.; Huwaimel, B.; Verma, K.; Miller, J.; Germain, T.M.; Kinarivala, N.; Pappas, D.; Brookes, P.S.; Trippier, P.C. Synthesis and Antineoplastic Evaluation of Mitochondrial Complex II (Succinate Dehydrogenase) Inhibitors Derived from Atpenin A5. *ChemMedChem* **2017**, *12*, 1033–1044, doi:10.1002/cmdc.201700196.

144. Soller, M.; Dröse, S.; Brandt, U.; Brüne, B.; von Knethen, A. Mechanism of Thiazolidinedione-Dependent Cell Death in Jurkat T Cells. *Mol Pharmacol* **2007**, *71*, 1535–1544, doi:10.1124/mol.107.034371.
145. Wang, L.; Zhang, X.; Cui, G.; Chan, J.Y.-W.; Wang, L.; Li, C.; Shan, L.; Xu, C.; Zhang, Q.; Wang, Y.; et al. A Novel Agent Exerts Antitumor Activity in Breast Cancer Cells by Targeting Mitochondrial Complex II. *Oncotarget* **2016**, *7*, 32054–32064, doi:10.18632/oncotarget.8410.
146. Fiorillo, M.; Lamb, R.; Tanowitz, H.B.; Mutti, L.; Krstic-Demonacos, M.; Cappello, A.R.; Martinez-Outschoorn, U.E.; Sotgia, F.; Lisanti, M.P. Repurposing Atovaquone: Targeting Mitochondrial Complex III and OXPHOS to Eradicate Cancer Stem Cells. *Oncotarget* **2016**, *7*, 34084–34099, doi:10.18632/oncotarget.9122.
147. Bhattacharya, K.; Bag, A.K.; Tripathi, R.; Samanta, S.K.; Pal, B.C.; Shaha, C.; Mandal, C. Mahanine, a Novel Mitochondrial Complex-III Inhibitor Induces G0/G1 Arrest through Redox Alteration-Mediated DNA Damage Response and Regresses Glioblastoma Multiforme. *Am J Cancer Res* **2014**, *4*, 629–647.
148. Chen, H.; Li, L.; Lu, Y.; Shen, Y.; Zhang, M.; Ge, L.; Wang, M.; Yang, J.; Tian, Z.; Tang, X. <p>Azoxystrobin Reduces Oral Carcinogenesis by Suppressing Mitochondrial Complex III Activity and Inducing Apoptosis</P>. *CMAR* **2020**, *12*, 11573–11583, doi:10.2147/CMAR.S280285.
149. Sun, R.C.; Board, P.G.; Blackburn, A.C. Targeting Metabolism with Arsenic Trioxide and Dichloroacetate in Breast Cancer Cells. *Mol Cancer* **2011**, *10*, 142, doi:10.1186/1476-4598-10-142.
150. Hescot, S.; Slama, A.; Lombès, A.; Paci, A.; Remy, H.; Leboulleux, S.; Chadarevian, R.; Trabado, S.; Amazit, L.; Young, J.; et al. Mitotane Alters Mitochondrial Respiratory Chain Activity by Inducing Cytochrome c Oxidase Defect in Human Adrenocortical Cells. *Endocr Relat Cancer* **2013**, *20*, 371–381, doi:10.1530/ERC-12-0368.
151. Shi, Y.; Lim, S.K.; Liang, Q.; Iyer, S.V.; Wang, H.-Y.; Wang, Z.; Xie, X.; Sun, D.; Chen, Y.-J.; Tabar, V.; et al. Gboxin Is an Oxidative Phosphorylation Inhibitor That Targets Glioblastoma. *Nature* **2019**, *567*, 341–346, doi:10.1038/s41586-019-0993-x.
152. Jung, K.-H.; Song, S.-H.; Paik, J.-Y.; Koh, B.-H.; Choe, Y.S.; Lee, E.J.; Kim, B.-T.; Lee, K.-H. Direct Targeting of Tumor Cell F1F0 ATP-Synthase by Radioiodine Angiostatin In Vitro and In Vivo. *Cancer Biotherapy and Radiopharmaceuticals* **2007**, *22*, 704–712, doi:10.1089/cbr.2007.369.
